# A Randomized, Observer-Blinded Immunogenicity Trial of Cervarix^®^ and Gardasil^®^ Human Papillomavirus Vaccines in 12-15 Year Old Girls

**DOI:** 10.1371/journal.pone.0061825

**Published:** 2013-05-01

**Authors:** Eve Draper, Sara L. Bissett, Rebecca Howell-Jones, Pauline Waight, Kate Soldan, Mark Jit, Nicholas Andrews, Elizabeth Miller, Simon Beddows

**Affiliations:** 1 Virus Reference Department, Health Protection Agency, London, United Kingdom; 2 HIV/STI Department, Health Protection Agency, London, United Kingdom; 3 Statistics, Modelling and Economics Department, Health Protection Agency, London, United Kingdom; 4 Immunisation, Hepatitis and Blood Safety Department, Health Protection Agency, London, United Kingdom; Aeras, United States of America

## Abstract

**Background:**

The current generation of Human Papillomavirus (HPV) vaccines, Cervarix® and Gardasil®, exhibit a high degree of efficacy in clinical trials against the two high-risk (HR) genotypes represented in the vaccines (HPV16 and HPV18). High levels of neutralizing antibodies are elicited against the vaccine types, consistent with preclinical data showing that neutralizing antibodies can mediate type-specific protection in the absence of other immune effectors. The vaccines also confer protection against some closely related non-vaccine HR HPV types, although the vaccines appear to differ in their degree of cross-protection. The mechanism of vaccine-induced cross-protection is unknown. This study sought to compare the breadth and magnitudes of neutralizing antibodies against non-vaccine types elicited by both vaccines and establish whether such antibodies could be detected in the genital secretions of vaccinated individuals.

**Methods and Findings:**

Serum and genital samples were collected from 12–15 year old girls following vaccination with either Cervarix® (n = 96) or Gardasil® (n = 102) HPV vaccine. Serum-neutralizing antibody responses against non-vaccine HPV types were broader and of higher magnitude in the Cervarix®, compared to the Gardasil®, vaccinated individuals. Levels of neutralizing and binding antibodies in genital secretions were closely associated with those found in the serum (r = 0.869), with Cervarix® having a median 2.5 (inter-quartile range, 1.7–3.5) fold higher geometric mean HPV-specific IgG ratio in serum and genital samples than Gardasil® (*p* = 0.0047). There was a strong positive association between cross-neutralizing antibody seropositivity and available HPV vaccine trial efficacy data against non-vaccine types.

**Conclusions:**

These data demonstrate for the first time that cross-neutralizing antibodies can be detected at the genital site of infection and support the possibility that cross-neutralizing antibodies play a role in the cross-protection against HPV infection and disease that has been reported for the current HPV vaccines.

**Trial Registration:**

ClinicalTrials.gov NCT00956553

## Introduction

Cancer of the cervix is the third most common cancer of women and accounts for an estimated 530,000 cervical cancer cases and 275,000 deaths per annum worldwide [1], [2]. Together, HPV16 and HPV18 are associated with *ca*. 70% of cervical cancer cases worldwide [3]–[5].

The current generation of virus-like particle (VLP)-based vaccines (Cervarix® and Gardasil®) target HPV16 and HPV18 and have been shown in clinical trials to be highly efficacious at reducing persistent infections and cervical lesions associated with these types [6], [7]. Following immunization, high levels of serum neutralizing antibodies against pseudovirions representing these two types and antibodies capable of blocking murine monoclonal antibodies targeting putative neutralizing domains on immobilized VLP can be detected [8]–[14]. These observations corroborate findings from animal models in which passive transfusion of immune sera or purified IgG protected animals from subsequent papillomavirus challenge [15], [16]. Taken together these data suggest that neutralizing antibodies against the vaccine types, HPV16 and HPV18, play a significant role in mediating vaccine-induced protection from infection and disease associated with these two HPV types [17]–[19].

It is not known whether neutralizing antibodies play a significant role in mediating cross-protection against non-vaccine types. Closely-related non-vaccine types within the Alpha-papillomavirus species groups A9 (HPV16-like: HPV31, HPV33, HPV35, HPV52, HPV58) and A7 (HPV18-like: HPV39, HPV45, HPV59, HPV68) [20], [21] are associated with a further *ca*. 25% of cervical cancer cases worldwide [3], [4]. The HPV vaccines have been shown to afford some degree of cross-protection against closely-related non-vaccine types HPV31, HPV33 and HPV45 but not against HPV52 or HPV58 [6], [7]. The vaccines appear to differ in their degree of cross-protection: Gardasil® does not seem to afford significant protection against HPV45 [6], [7]. Immunization with Cervarix® vaccine elicits neutralizing antibodies against a range of HPV types from the A7 and A9 species groups and the patterns of antibody recognition appear to be at least coincident with available protection data [8], [10]. There are no comparable data from Gardasil® vaccinees.

The purpose of this study was to compare the ability of the HPV vaccines (Cervarix® and Gardasil®) to elicit cross-neutralizing antibodies against all HR HPV types [22] phylogenetically related to the vaccine types and to establish whether cross-neutralizing antibodies could be detected in the genital secretions of vaccinated individuals, a prerequisite for ascertaining whether such antibodies play a role in vaccine-induced cross-protection.

## Materials and Methods

The protocol for this trial and supporting CONSORT checklist are available as supporting information (Protocol S1 and Checklist S1).

### Ethics statement

Ethical Approval was obtained from the UK National Research Ethics Service (Research Ethics Committee reference 09/H0720/25). The study protocol was approved by the UK Medicines and Healthcare products Regulation Agency (MHRA) and registered on the *ClinicalTrials.gov* website (NCT00956553) prior to subject recruitment. The study was conducted in accordance with the Declaration of Helsinki and Good Clinical Practice guidelines.

### Study design, immunization schedule and sample collection

This was a Phase IV trial conducted in two regions in England: Gloucestershire and Hertfordshire. Inclusion criteria were (i) 12–15 year old girls; (ii) written informed consent from a parent or guardian of the subject. Exclusion criteria were (i) already received or were currently receiving HPV vaccination; (ii) pregnant or become pregnant during the study; (iii) breast-feeding mothers; (iv) allergic to vaccine components (Protocol S1). A computerised block randomisation list was produced with each vaccine research nurse allocated blocks of sequential numbers in accordance with the block size used for randomisation. The first subject was enrolled during October 2009 and the last subject was enrolled during November 2010. This process took longer than the 10 months projected timeframe (Protocol S1). The final sample was collected December 2011. On recruitment to the study, each subject was allocated, in order of inclusion, the next available study number. Participants were thus randomized (1 1) to receive three doses of either the bivalent (Cervarix®; [23]) or quadrivalent (Gardasil®; [24]) HPV vaccine at Month (M) 0, 1 and 6. This is the recommended dosing schedule for Cervarix® and within the flexibility of the dosing schedule for Gardasil®. The immunogenicity of the HPV6 and HPV11 components of the quadrivalent vaccine were not studied as Cervarix® does not contain these two HPV types.

Blood samples were collected at M0 (prior to vaccination), M2 (one month post second dose), M7 (one month post third dose) and M12 (six months post third dose) and shipped to the testing laboratory (HPA, London) for serum separation and subsequent storage at −80°C. At M7 an optional self-taken lower vaginal swab sample (Netcell Slimpack™ Polyvinyl acetate media; Network Medical Products, UK) was collected, placed in a sterile dry universal container and shipped at +4°C to the testing laboratory.

The study was conducted in a blinded manner such that the laboratory staff and the subjects receiving the vaccine were unaware of the vaccine used until after completion of the laboratory testing (laboratory staff) or dosing schedule (subjects). Subject demographic and reactogenicity data were collected on case report forms and double entered and verified using an MS Access database. Laboratory results were imported into the study database and matched using a unique subject number.

### Antibody isolation from genital samples

Genital swab samples were weighed, rehydrated with 3 mL ice cold phosphate buffered saline (PBS; pH 7.4) containing 0.5% foetal bovine serum for 30 minutes on ice with agitation and subjected to centrifugation within a 50 mL Amicon tube (30 kDa cutoff; Millipore, UK) for 5 minutes at 2,500×g. Two such extractions were performed for each sample and the eluted material pooled and subjected to centrifugation at 13,000×g to remove cellular debris. The clarified supernatant was then made into single use aliquots and stored at −80°C. Genital samples were evaluated for the presence of neutralizing and L1 VLP binding antibodies against vaccine (HPV16, HPV18) and non-vaccine (HPV31, HPV45) types.

### Detection of IgG levels in serum and genital samples

Human IgG levels were estimated in serum and genital samples using an indirect ELISA. Goat anti-human IgG (100 ng; Invitrogen, UK) antibody was immobilized overnight onto 96-well Nunc-Immuno™ Maxisorp plates (Thermo Scientific, UK) before being blocked with 5% skimmed milk powder in PBS. Study samples and a set of human IgG standards (250–10 ng/mL; Invitrogen) were diluted in 0.5% skimmed milk powder in PBS and incubated at 37°C for 1.5 hours. Captured human IgG was resolved using goat anti-human IgG horseradish peroxidise (HRP) conjugate (Invitrogen), 3,3′,5,5′- tetramethylbenzidine (TMB) substrate (Thermo Scientific) and the Glomax Multi Detection System (450 nm λ) (Promega, UK). The linearity of the standards used was good with a median *r^2^* of 0.992 (Inter-quartile range, IQR, 0.986–0.997; n = 6).

### Pseudovirus neutralization assay

The HPV pseudovirus neutralization assay [25] was carried out as previously described using the extended set of A7 and A9 psheLL L1L2 pseudovirus clones [8]. Bovine Papillomavirus (BPV) was used as a control for non-specific antibody effects. Inter-assay reproducibility of neutralizing antibody titers was demonstrated by including in every experiment a control serum pool made with sera from Cervarix® vaccinees [8]. The geometric mean titer (GMT) of the control serum pool were as follows: HPV16 59,903 (95% CI, 49,479–72,523; n = 33), HPV18 29,863 (23,514–37,926; 34), HPV31 200 (156–256; 29), HPV33 31 (24–39; 17), HPV35 21 (17–27; 16), HPV39 11 (10–12; 19), HPV45 32 (26–40; 24), HPV52 23 (19–28; 21), HPV58 103 (86–124; 19), HPV59 11 (10–13; 16), HPV68 11(10–13; 14) and BPV 10 (10–10; 19). Heparin (H-4784; Sigma-Aldrich, UK) was also included as a positive inhibitor control. A small number of serum samples used in this study (n = 19; 10% of total) were also retested against nine pseudoviruses (HPV16, HPV18, HPV31, HPV33, HPV35, HPV39, HPV45, HPV52 and HPV58). The linearity of the resulting paired titers (n = 171) was very good (*r ^2^* = 0.989).

### VLP ELISA

For the detection of binding antibodies, L1 VLP were expressed using the Bac-to-Bac® Baculovirus Expression System (Invitrogen) and Sf21 insect cells as described previously [26]. Amino acid sequences for HPV16 (DQ469930), HPV18 (AY262282), HPV31 (J04353) and HPV45 (X74479) VLP shared 100% homology with their respective pseudovirus L1 sequence and the indicated GenBank accession numbers. The L1 concentration and purity were visualized by SDS PAGE stained with SimplyBlue™ Safestain (Invitrogen) and analyzed using ImageJ software (http://imagej.nih.gov/ij; National Institutes of Health, USA). VLP formation was confirmed by electron microscopic analysis of negatively staining particles (Phosphotungstic Acid; Sigma-Aldrich) adsorbed on copper grids coated with formvar (Sigma-Aldrich) and carbon.

VLP (25 ng) were immobilized onto 96-well Nunc-Immuno™ Polysorb plates (Thermo Scientific) before being blocked with 5% normal sheep serum (NSS) (AbCam, UK) and 5% non-fat milk diluted in Tris Buffered saline (TBS). Study sera, genital samples and control reagents were diluted in 20% NSS, 2% non-fat milk and 0.05% TWEEN® 20 (Sigma-Aldrich) in TBS and incubated for 1 hour at 37°C before resolution of binding was made by addition of a goat anti-human alkaline phosphatase conjugated antibody (Invitrogen) and the NAPDH-based ELISA amplification system (Invitrogen) and the GloMax Multi Detection System (490 nm λ; Promega, UK). Midpoint binding titers were estimated by interpolation. The high HPV16/18 and HPV negative plasma pools [27] were used as positive and negative serological control reagents, respectively. The high HPV16/18 reagent gave median (IQR) titers against the indicated HPV types as follows (n = 5): HPV16 69,124 (64,602–73,962), HPV18 41,211 (39,270–43,247), HPV31 2,655 (1,772–3,979) and HPV45 4,918 (4,452–5,432). The HPV negative reagent was negative in all tests.

### Statistical methods

The target sample size was 200 in each group. This would provide precision (95% CI width) of at most ±7% for estimation of percentages positive and allow differences of about 10 to 15% to be detectable between groups with 80% power at a 5% significance level. Despite an extended recruitment window, the recruited sample size was approximately half of the intended number. This limited the precision of estimates to about ±10% for 95% CIs and detectable differences between groups to about 15 to 20%.

Analysis was per-protocol, with major protocol deviations leading to removal of individuals or sample results. Major protocol deviations included an interval of -1 week to +3 weeks between doses 1 and 2 outside of the intended one month scheduled time and −4 weeks to +8 weeks between doses 2 and 3 outside of the intended five months scheduled time. Samples with missing results were omitted and those with censored results given a value of half the censoring value (for example, neutralization titers less than the lower limit of detection, <20, were set to 10).

At each time point, the proportion with titres ≥20 are given with exact 95% CI and the overall GMTs are given with 95% CI. Proportions were compared by Fisher's exact test. Comparison between study arms was made by the Wilcoxon rank-sum (Mann Whitney U) test. The non-parametric test for trend was used to assess the difference between non-vaccine type neutralization data ordered into tertiles based upon neutralizing antibody titers against the appropriate, HPV16 or HPV18, vaccine type.

For vaccinees with detectable antibodies in both serum and genital secretions, the HPV-specific antibody titer was normalized to the total IgG level in the serum or genital sample, respectively, and reported as the HPV specific IgG/total IgG serum. Following a test for normality (Shapiro-Wilk test), supported by a normal quantile (Q-Q) plot, Pearson's correlation (*r*) was used to evaluate the relationship between the Log_10_ normalized values for these two sample types [28].

The linear association between vaccine efficacy (against CIN2+ and persistent infection) as reported from clinical trials [29], [30] and the seroprevalence (neutralizing antibody) data generated in this study was investigated. To propagate the uncertainty in these variables to the uncertainty in the linear relationship, 1000 bootstrap samples with replacement were taken of the populations of individuals in the studies used to estimate efficacy and seroprevalence [31]. These were used to construct 1000 bootstrap estimates of vaccine efficacy and seroprevalence. Each estimate of vaccine efficacy was paired with one of seroprevalence, and related by a separate linear regression; the uncertainty in the graphs represents the 95% range of predictions across the models. Exact 95% confidence intervals for data points were calculated using the Fisher's exact method for vaccine efficacy (1-odds ratio) and Clopper-Pearson for seroprevalence.

Significance was taken at the 5% level and 95% confidence intervals used. Two-sided significance tests were used. Stata version 12 (StataCorp, USA) was used for the analyses.

## Results

### Subjects enrolled

**Figure 1:**
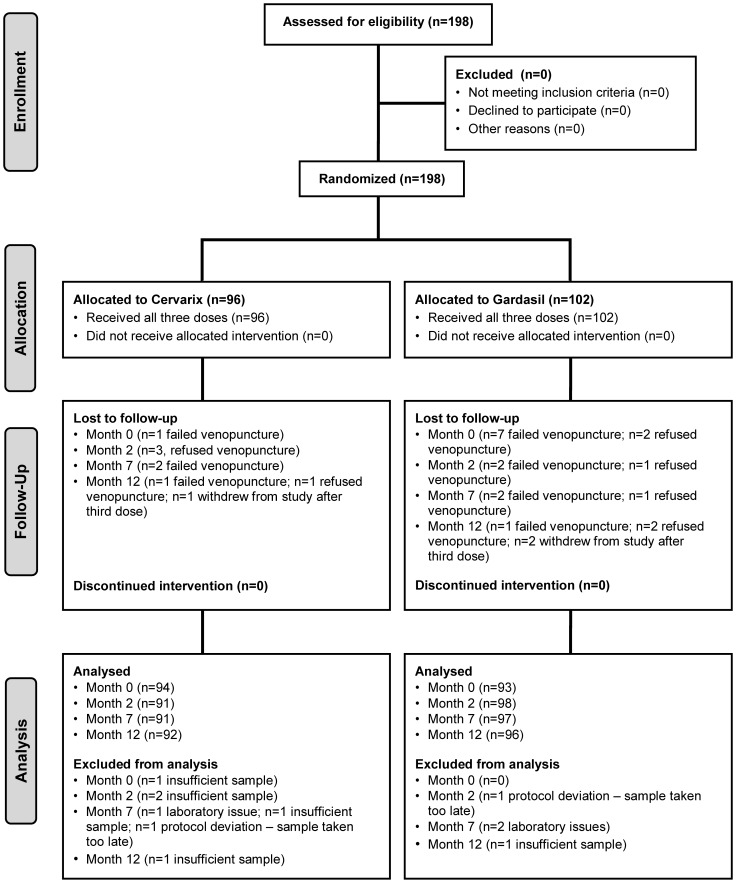
CONSORT diagram. Allocations, interventions, follow up and analysis of 198 subjects recruited into the comparative HPV vaccine immunogenicity study.

One hundred and ninety eight subjects were enrolled and randomly allocated to receive either Cervarix® (n = 96) or Gardasil® vaccine (n = 102). All individuals received three vaccine doses although there were a small number of samples lost to follow up and/or excluded from the analysis (**Figure 1**). The ages of recruited individuals to both study arms were similar at a median 12 (range 12–15) years old. The implemented vaccination schedule was close to the planned schedule and similar for both study groups at a median of 28 days between the first and second vaccine doses and a median 154 days between the second and third vaccine doses.

### Safety

Information on vaccine reactogenicity was provided by all participants. The percentages of girls reporting at least one solicited symptom within seven days of any vaccine dose are reported in **Table S1**. Injection site pain was the most frequently reported symptom in 93.8% of Cervarix® vaccinees compared to 86.3% of Gardasil® vaccinees (*p*>0.05); 24% of Cervarix® vaccinees reported an incident of moderate or severe pain which was higher than the 6.9% reported for the Gardasil® vaccinees (*p* = 0.001). Local swelling and redness were commonly reported, but these reactions were mild (<50 mm) and similar between the vaccines (*p*>0.05). Approximately 10–20% of participants reported a moderate or severe general episode (including loss of appetite, fatigue, and headache) but these were similar between the vaccines (*p*>0.05). Approximately, one fifth of participants within each vaccine arm (*p*>0.05) reported an elevated temperature (≥37.5°C) within a 7 day period of receiving a vaccine dose but only in one case (a Gardasil® vaccinee) was this considered severe (≥39°C). Overall, the vaccine schedule was well tolerated.

### Baseline HPV antibody reactivity

M0 serum samples from the Cervarix® (n = 94) and Gardasil® (n = 93) vaccine arms were tested against HPV pseudoviruses within the A7 and A9 species groups and the control BPV (**Table 1**). A small number of individuals demonstrated antibody reactivity against HPV16 (n = 3; titers 22, 27, 95), HPV31 (n = 1; 154), HPV33 (n = 2; 20, 21), HPV35 (n = 1; 61), HPV58 (n = 1; 809), HPV18 (n = 1; 24) and HPV68 (n = 1; 26) consistent with a small number of natural infections in this age group and potentially highlighting the limit of assay specificity for some pseudoviruses. The exception to this was pseudovirus HPV52 for which a high proportion of reactive samples was seen for both Cervarix® (n = 30; 31.9%) and Gardasil® (n = 37; 39.8%) with corresponding GMTs of 16 (95% CI, 14–18) and 17 (15–20) for the Cervarix® and Gardasil® vaccine, respectively. For calculation purposes we assumed that all individuals were HPV antibody negative at M0, although this is likely to give a slight underestimate of the true assay specificity. On this basis, overall assay specificity was 96.6% (95% CI, 95.7–97.3) while assay specificity excluding HPV52 data was 99.5% (95% CI, 99.1–99.8).

**Table 1:** Seropositivity for neutralizing antibodies against A9 and A7 pseudoviruses at entry (Month 0).^a^

		Cervarix®		Gardasil®	
Clade	HPV	n	% (95% CI)	n	% (95% CI)
A9	16	0	0.0% (0.0–4.0)	3	3.0% (1.0–9.0)
	31	1	1.1% (0.0–5.8)	0	0.0% (0.0–3.9)
	33	0	0.0% (0.0–3.8)	2	2.2% (0.3–7.6)
	35	0	0.0% (0.0–3.8)	1	1.1% (0.0–5.8)
	52	30	31.9% (22.7–42.3)	37	39.8% (29.8–50.5)
	58	1	1.1% (0.0–5.8)	0	0.0% (0.0–3.9)
A7	18	1	1.1% (0.0–5.8)	0	0.0% (0.0–3.9)
	39	0	0.0% (0.0–3.8)	0	0.0% (0.0–3.9)
	45	0	0.0% (0.0–3.8)	0	0.0% (0.0–3.9)
	59	0	0.0% (0.0–3.8)	0	0.0% (0.0–3.9)
	68	0	0.0% (0.0–3.8)	1	1.1% (0.0–5.8)
Control	BPV	0	0.0% (0.0–3.8)	0	0.0% (0.0–3.9)

a Cervarix n = 94; Gardasil n = 93

### Neutralizing antibody titers following vaccination

**Figure 2:**
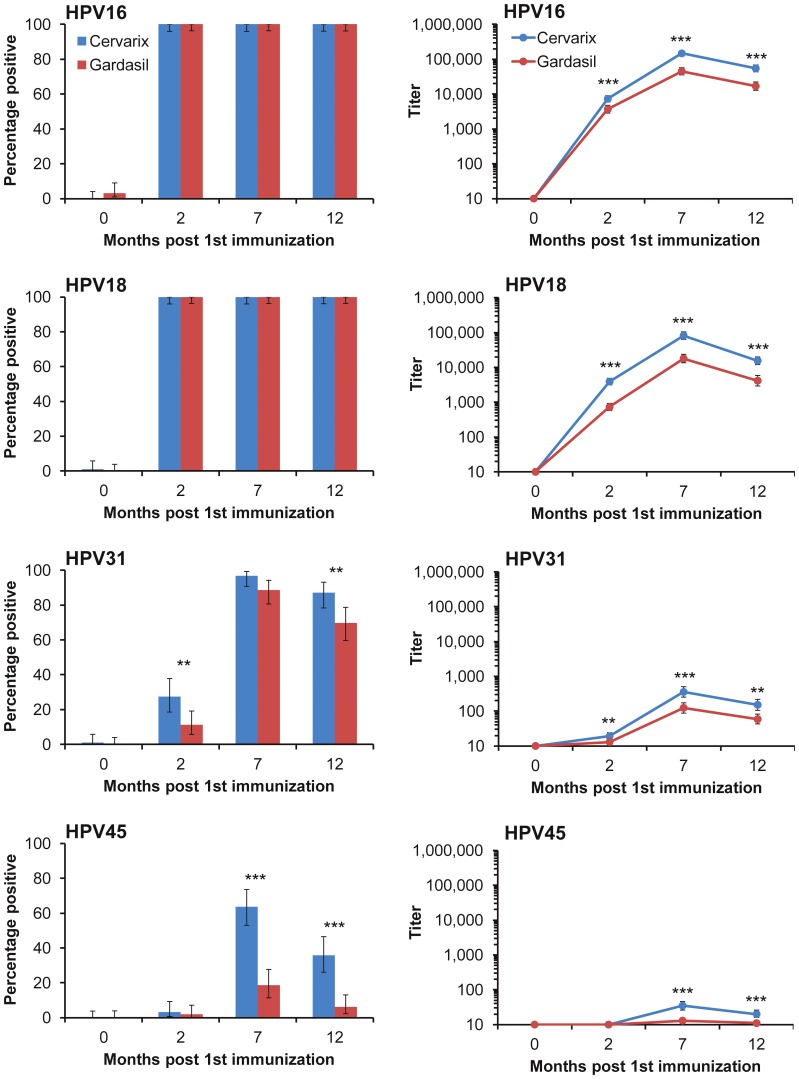
Neutralizing antibody responses over time course of study. Percentage of vaccinees with neutralization titers of ≥20 (left panels) and GMT (right panels) against indicated vaccine (HPV16, HPV18) and non-vaccine (HPV31, HPV45) types. Error bars, ±95% CI. * *p*<0.05; ** *p*<0.01; *** *p*<0.001. Denominators for each measure were as follows: Cervarix® (blue) M0 (n = 94), M2 (n = 91), M7 (n = 91), M12 (n = 92); Gardasil® (red) M0 (n = 93), M2 (n = 98), M7 (n = 97), M12 (n = 96).

As expected, all individuals generated high titer neutralizing antibodies against the vaccine types, HPV16 and HPV18, following three doses (M7) of either Cervarix® or Gardasil® (**Table 2**). Antibody responses against HPV16 were of a higher magnitude than those against HPV18 for both Cervarix® (median 1.6 [inter-quartile range, IQR, 0.9–3.3] fold; n = 91; *p*<0.001) and Gardasil® (2.2 [IQR, 1.3–5.6] fold; n = 97; *p*<0.001) and responses generated following immunization with Cervarix® were higher than those obtained for Gardasil® for both HPV16 and HPV18 (*p*<0.001). These differences were consistent throughout the time course under study (**Figure 2**). For example, one month following the second vaccine dose (M2), while 100% of vaccinees were positive for neutralizing antibodies against HPV16, the GMT in the Cervarix® arm was 7,284 (95% CI, 5,920–8,963) compared to 3,685 (95% CI, 2,844–4775) in the Gardasil® arm (*p*<0.001).

**Table 2:** Peak seropositivity and geometric mean neutralizing antibody titers against A9 and A7 HPV pseudoviruses (Month 7).^a^

		Percentage seropositive					Geometric mean titers		
		Cervarix®		Gardasil®			Cervarix®	Gardasil®	
Clade	HPV	n	% (95% CI)	n	% (95% CI)	p value	GMT (95% CI)	GMT (95% CI)	p value
A9	16	91	100% (96.0–100)	97	100% (96.3–100)	1.000	146,979 (123,167–175,394)	45,220 (35,573–57,485)	**<0.001**
	31	88	96.7% (90.7–99.3)	86	88.7% (80.6–94.2)	0.050	356 (253–502)	124 (88–174)	**<0.001**
	33	67	73.6% (63.3–82.3)	51	52.6% (42.2–62.8)	**0.004**	44 (34–56)	28 (22–36)	**0.009**
	35	35	38.5% (28.4–49.2)	11	11.3% (5.8–19.4)	**<0.001**	20 (16–26)	12 (11–13)	**<0.001**
	52	78	85.7% (76.8–92.2)	60	61.9% (54.4–71.5)	**<0.001**	53 (43–66)	25 (21–30)	**<0.001**
	58	35	38.5% (28.4–49.2)	19	19.6% (12.2–28.9)	**0.001**	17 (14–19)	13 (11–14)	**0.005**
A7	18	91	100% (96.0–100)	97	100% (96.3–100)	1.000	81,434 (63,352–104,676)	17,907 (13,537–23,689)	**<0.001**
	39	4	4.4% (1.2–10.9)	2	2.1% (0.3–7.3)	0.430	12 (10–14)	10 (10–11)	0.350
	45	58	63.7% (53.0–73.6)	18	18.6% (11.4–27.7)	**<0.001**	35 (26–46)	13 (11–14)	**<0.001**
	59	1	1.1% (0.0–6.0)	0	0.0% (0.0–3.7)	0.480	10 (10–11)	10 (10–10)	0.300
	68	3	3.3% (0.7–9.3%)	1	1.0% (0.0–5.6)	0.360	10 (10–11)	10 (10–10)	0.280
Control	BPV	0	0.0% (0.0–4.0)	0	0.0% (0.0–3.7)	1.000	10 (10–10)	10 (10–10)	N/A

a Cervarix n = 91; Gardasil n = 97. *p* values <0.05 highlighted in bold type. N/A, not applicable

Sera able to neutralize non-vaccine HPV types from the A9 species group (HPV31, HPV33, HPV35, HPV52 and HPV58) were common amongst these vaccinees (**Table 2**). Neutralizing antibody responses following vaccination with Cervarix® were consistently more frequent and of higher titer than those obtained following vaccination with Gardasil®. Antibody responses against non-vaccine HPV types from the A7 species group were largely limited to those against HPV45. Cervarix® vaccinees generated higher titer responses against HPV31 and HPV45 than Gardasil® and these differences were consistent across the time course under study (**Figure 2**). The proportion of responders and the resulting GMTs against HPV31 and HPV45 were substantially higher after a third dose of vaccine (M7) compared to responses following two doses (M2) and this was apparent for both vaccines. No cross-reactivity against the control BPV was observed.

### Association of vaccine and non-vaccine type antibody responses

The relationship between vaccine and non-vaccine specific neutralizing antibody responses was investigated by categorizing the sera into tertiles based on the vaccine-type titers for each species group (HPV16 tertiles for A9 types and HPV18 tertiles for A7 types) (**Table 3**) [8], [10]. Antibody responses against non-vaccine types HPV31, HPV33, HPV35, HPV45 and HPV52 increased in line with vaccine-type responses for both vaccines. For example, 93% of sera in the lowest HPV16 tertile of Cervarix® vaccinees were positive for antibodies against HPV31, rising to 100% in the highest tertile. The GMTs against HPV31 increased from 121, to 375, to 993 in the lowest, middle and highest tertiles, respectively (*p*<0.001). Antibody responses against HPV58 (Gardasil® only) and HPV39 (Cervarix® only) also demonstrated some degree of association with their vaccine-type responses.

**Table 3:** Evaluation of cross-neutralizing antibody responses in relation to type-specific titers.^a^

			Cervarix®			Gardasil®		
Clade	HPV	Tertile	n (%)	GMT (95% CI)	*p* value	n (%)	GMT (95% CI)	*p* value
A9	31	Low	28 (93)	121 (84–173)		22 (69)	41 (25–66)	
		Middle	30 (97)	375 (214–656)		32 (97)	96 (61–151)	
		High	30 (100)	993 (532–1,854)	**<0.001**	32 (100)	491 (292–828)	**<0.001**
	33	Low	17 (57)	24 (17–33)		8 (25)	15 (11–21)	
		Middle	23 (74)	46 (29–72)		17 (52)	25 (17–36)	
		High	27 (90)	77 (50–118)	**<0.001**	26 (81)	61 (38–99)	**<0.001**
	35	Low	8 (27)	14 (11–18)		0 (0)	10 (10–10)	
		Middle	12 (39)	23 (14–39)		5 (15)	12 (10–15)	
		High	15 (50)	25 (16–38)	**0.042**	6 (19)	13 (11–15)	**0.020**
	52	Low	23 (77)	38 (27–52)		14 (44)	20 (14–28)	
		Middle	26 (84)	52 (36–75)		21 (64)	23 (18–29)	
		High	29 (97)	76 (48–119)	**0.021**	25 (78)	35 (26–48)	**0.006**
	58	Low	8 (14)	15 (11–20)		2 (6)	11 (9–13)	
		Middle	14 (45)	19 (14–25)		6 (18)	13 (10–16)	
		High	13 (43)	16 (13–20)	0.265	11 (34)	15 (12–18)	**0.008**
A7	39	Low	0 (0)	10 (10–10)		1 (3)	10 (10–12)	
		Middle	0 (0)	10 (10–10)		0 (0)	10 (10–10)	
		High	4 (13)	16 (9–30)	**0.012**	1 (3)	10 (10–11)	0.986
	45	Low	11 (37)	19 (13–28)		1 (3)	10 (10–11)	
		Middle	24 (77)	39 (26–58)		8 (24)	14 (11–17)	
		High	23 (77)	55 (30–101)	**0.001**	9 (28)	14 (11–18)	**0.011**
	59	Low	0 (0)	10 (10–10)		0 (0)	10 (10–10)	
		Middle	1 (3)	11 (9–15)		0 (0)	10 (10–10)	
		High	0 (0)	10 (10–10)	1.000	0 (0)	10 (10–10)	N/A
	68	Low	1 (3)	11 (9–12)		1 (3)	10 (10–11)	
		Middle	0 (0)	10 (10–10)		0 (0)	10 (10–10)	
		High	2 (7)	11 (10–12)	0.492	0 (0)	10 (10–10)	0.218

a Tertiles based on distribution of type-specific neutralization titers for Cervarix® (low tertile, n = 30; middle tertile, n = 31; high tertile, n = 30) and Gardasil® (low tertile, n = 32; middle tertile, n = 33; high tertile, n = 32). Within tertile vaccine type responses for HPV16 (Cervarix® Low: GMT 59,389 [95% CI, 49,560–71,166]; Middle: 141,590 [130,070–154,130]; High: 378,067 [324,004–441,151] and Gardasil® Low: 12,739 [9,962–16,291]; Middle 45,446 [39,644–52,096]; High: 159,697 [124,661–204,580]) and HPV18 (Cervarix® Low: 21,847 [16,844–28,335]; Middle: 85,628 [79,238–92,533]; High: 288,194 [219,390–378,576] and Gardasil® Low: 3,971 [2,827–5,578]; Middle 18,877 [16,876–21,114]; High: 76,482 [59,947–97,576]) were as indicated. n (%), number of sera positive (titer ≥20) for neutralizing antibody against indicated HPV type and, in parentheses, percentage of total samples in each tertile. *p* value generated by trend analysis with significant associations highlighted in bold type. GMT (95% CI), geometric mean titer (95% confidence intervals in parentheses); for calculation purposes a titer <20 was assigned a value of 10. N/A, not applicable

**Figure 3:**
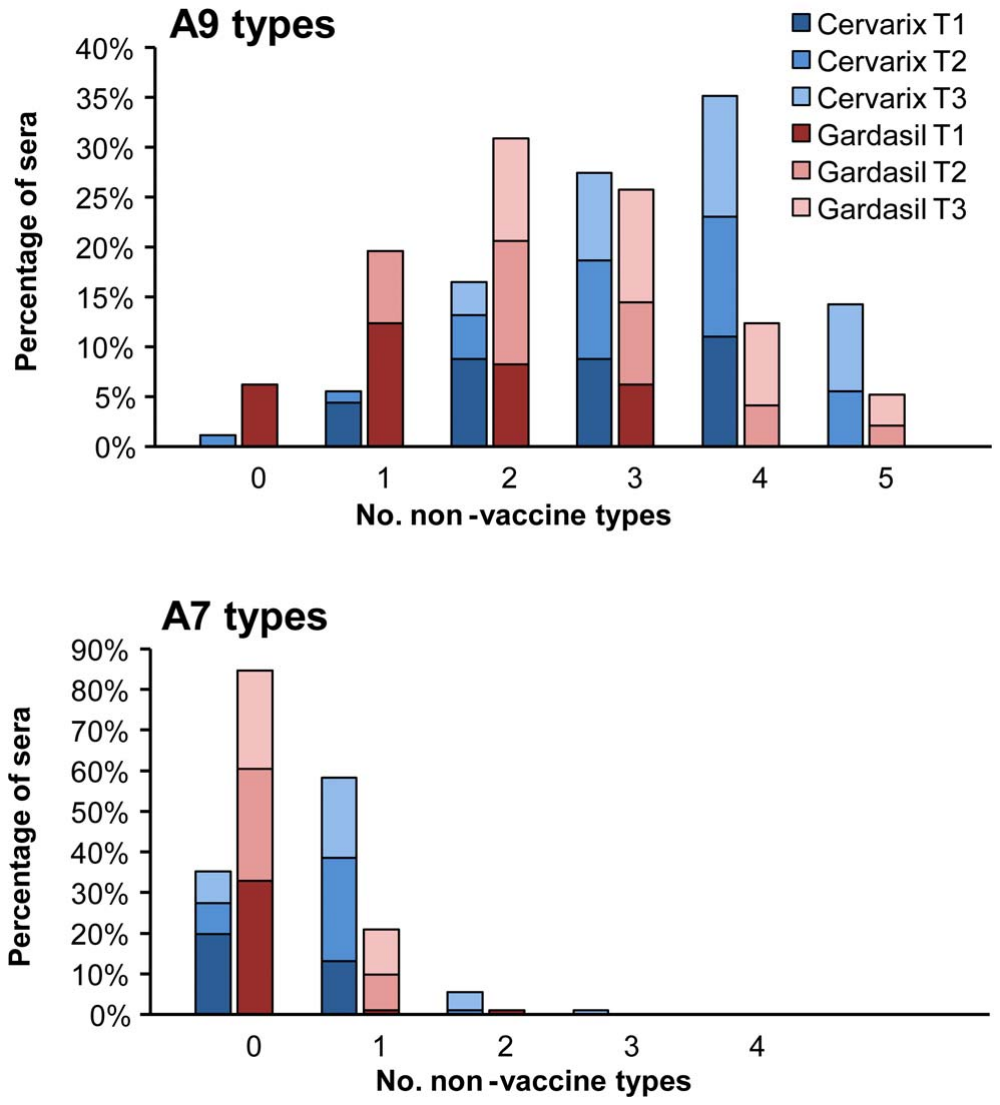
Breadth of neutralizing antibody responses elicited by HPV vaccines. Percentage of serum samples neutralizing indicated number of A9 (top panel) or A7 (bottom panel) non-vaccine types within each of the low (T1), middle (T2) or high (T3) vaccine-type tertiles (dark to light gradient shading) for indicated vaccine (blue, Cervarix®; red, Gardasil®).

The number of non-vaccine HPV types neutralized by each serum generally increased with vaccine-type titer such that broadly reactive sera tended to be those segregated into the highest tertiles (**Figure 3**). This was apparent for responses against viruses within both the A9 (for either vaccine, *p*<0.001) and A7 (Cervarix®, *p* = 0.001; Gardasil®, *p* = 0.028) species groups. Cervarix® vaccinees also generated an overall broader antibody response than Gardasil® vaccinees (*p*<0.001) (**Tables 2** and **3**).

### Comparison of vaccine and non-vaccine type responses in serum and genital secretions

Around 25% of participants consented to provide a self-taken lower genital swab at M7: 21 from the Cervarix® arm and 29 from the Gardasil® arm. The geometric mean of the recovered IgG from these genital secretion samples was 141 (95% CI, 108–185) µg/mL.

All genital samples in the Cervarix® study arm were positive for neutralizing antibodies against the vaccine types (HPV16 n = 21, GMT 369 [95% CI, 191–713]; HPV18 n = 21, 219 [119–401]) as were most samples in the Gardasil® study arm (HPV16 n = 28, 124 [63–242]; HPV18 n = 26, 56 [30–106]). Genital swab titers in the Cervarix® arm were higher than in the Gardasil® arm for both HPV16 (*p* = 0.028) and HPV18 (*p* = 0.005). All genital samples were positive for antibodies against HPV16 or HPV18 VLP with higher titers (*p*≤0.001) seen for Cervarix® (HPV16 GMT 1,479 [95% CI, 662–3,304]; HPV18 801 [402–1,598]) than for Gardasil® (HPV16 475 [311–724]; HPV18 308 [206–460]).

**Figure 4:**
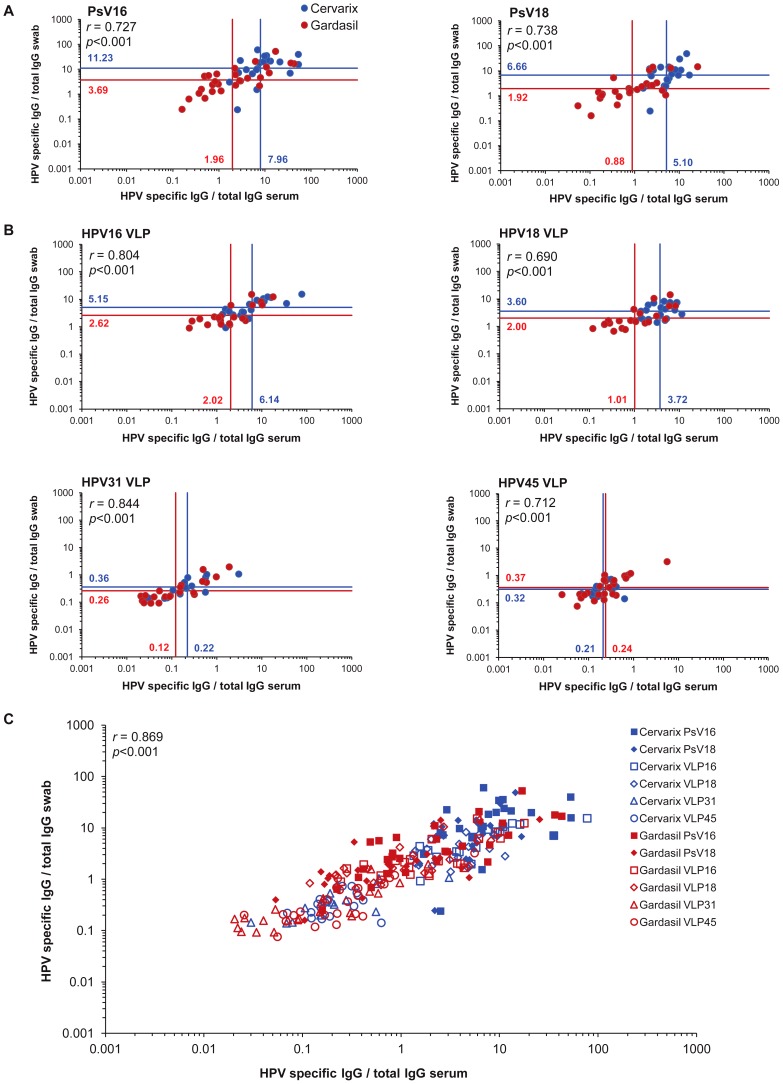
Relationship between HPV-specific vaccine antibodies in sera and genital samples. (A) Neutralization titers were normalized to IgG levels in serum and genital samples for HPV16 (left panel; Cervarix® n = 21, Gardasil® n = 28) and HPV18 (right panel; Cervarix® n = 21, Gardasil® n = 26). (B) VLP binding titers were normalized to IgG levels in serum and genital samples for HPV16 (top left panel; Cervarix® n = 20, Gardasil® n = 21), HPV18 (top right panel; Cervarix® n = 19, Gardasil® n = 21), HPV31 (bottom left panel; Cervarix® n = 14, Gardasil® n = 21) and HPV45 (bottom right panel; Cervarix® n = 17, Gardasil® n = 24). Geometric mean ratios of the HPV specific IgG/total IgG for each sample type presented for Cervarix® (blue lines) and Gardasil® (red lines). (C) Relationship between serum and genital samples using normalized neutralizing and binding antibody for all tests. Overall Pearson's (*r*) coefficients are given. PsV, HPV pseudovirus. VLP, HPV virus-like particle.

As the measurement of genital HPV-specific antibody levels is strictly dependent on the efficiency of antibody recovery, the presentation of antibody titers (as a function of the eluted sample dilution) is not particularly informative. Instead, neutralizing and binding antibody levels were normalized to the sample IgG concentration. The geometric mean of the vaccine-type HPV-specific IgG ratios found in the sera were closely related to those found in the genital secretions and this was apparent for both neutralizing (HPV16 *r* = 0.727, HPV18 *r* = 0.738; **Figure 4A**) and VLP binding (HPV16 *r* = 0.804, HPV18 *r* = 0.690; **Figure 4B**) antibodies for both vaccine arms.

Overall, only 20% (n = 10) and 4% (n = 2) of swabs contained sufficient amounts of recoverable antibody to be detected in neutralization tests against HPV31 and HPV45, respectively. This was not surprising given the very low titers detected against HPV31 and HPV45 in serum and the generally low levels of recoverable IgG in genital samples. There were, therefore, too few genital samples positive for neutralizing antibodies against these non-vaccine types to evaluate the linearity of the relationship between the serum and the genital secretions, as was done for HPV16 and HPV18. However, all serum samples and the majority of the genital samples were positive for binding antibodies against HPV31 and HPV45 VLP and demonstrated a good relationship between the HPV31 or HPV45-specific IgG in serum and genital samples (HPV31 *r* = 0.844, HPV45 *r* = 0.712; **Figure 4B**).

The combined geometric mean HPV-specific IgG neutralizing (HPV16, HPV18) and binding (HPV16, HPV18, HPV31 and HPV45) ratios in serum and genital samples was a median 2.5 (IQR, 1.7–3.5) fold higher in Cervarix® vaccinees compared to Gardasil® vaccinees (*p* = 0.0047). The relationship between the HPV-specific IgG in serum and genital samples, using the neutralizing and binding data from all tests (n = 253 pairs), was very good (*r* = 0.869; **Figure 4C**).

Of the genital samples positive for neutralizing antibodies against HPV31, representative plots of the neutralizing and binding antibody profiles against HPV16, HPV18 and HPV31 are shown in **Figure S1**. These highlight the close relationship between the titration curves against each HPV type, including the non-vaccine type HPV31, for the serum and genital sample responses when normalized to the IgG concentrations of each sample.

**Figure 5:**
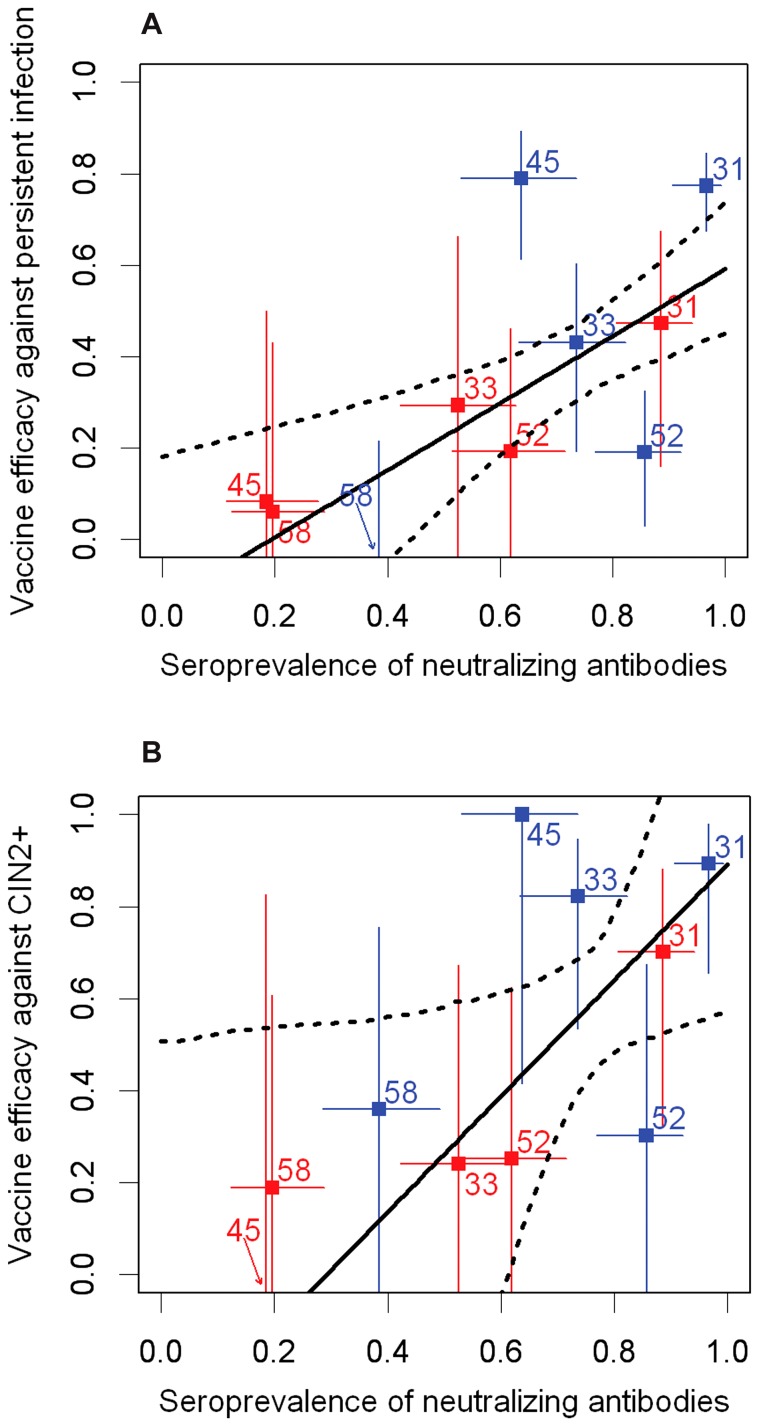
Type-specific neutralizing antibody seroprevalence in study participants against reported vaccine efficacy. Seroprevalence data are plotted against (A) efficacy against persistent infection and (B) efficacy against CIN2+ reported from trials of Cervarix® (blue points) and Gardasil® (red points) vaccinees for each non-vaccine HPV type (HPV31, HPV33, HPV45, HPV52 and HPV58). Vertical error bars represent exact 95% confidence intervals for the efficacy data, while horizontal error bars represent exact 95% confidence intervals for the neutralization seroprevalence data. The best fitting linear relationship between the two variables (black line) and the 95% range of bootstrap estimates for this relationship (dotted lines) is also shown. Exact 95% confidence intervals for data points were calculated using the Fisher's exact method for vaccine efficacy (1-odds ratio) and Clopper-Pearson for seroprevalence.

Finally, we wanted to compare the neutralizing antibody seroprevalence to non-vaccine types in our study participants (at month 7 after vaccination) with the available HPV vaccine efficacy against CIN2+ and persistent infection reported from clinical trials (on average 3.5 years after vaccination). This comparison shows an apparent strong positive association between seroprevalence and efficacy, with mean line coefficients of 0.73 (95% range 0.32–1.3) and 1.2 (95% range 0.22–3.5) for the association with efficacy against persistent infection (**Figure 5A**) and CIN2+ (**Figure 5B**) respectively.

## Discussion

This study attempted to evaluate the breadth and magnitude of neutralizing antibodies elicited by both currently available HPV vaccines, using pseudovirus clones representing non-vaccine HR HPV types, in order to address whether differences in the antibody responses seen would parallel the differences in cross-protection reported from clinical trials of Cervarix® and Gardasil®. We also wished to ascertain whether cross-neutralizing antibodies could be detected in genital secretions, a prerequisite for investigating neutralizing antibodies as a mediator of cross-protection.

Participants were of the target age group for HPV immunization in the UK and younger than the 18-26 year old females enrolled into the only other published study (HPV-010) to examine the immunogenicity of both HPV vaccines in parallel [12], [32]. All participants received all three doses of their allocated vaccine and the immunization regimen was well tolerated. Injection site pain the most common post-vaccination symptom and this was reported more frequently for Cervarix® than for Gardasil® vaccinees. This appears to be a common finding for the Cervarix® vaccine [12]. In order to align the schedules for both vaccines and maintain observer blindness we reduced the second Gardasil® dose from month 2 to month 1, in keeping with the recommended flexibility for this vaccine. We do not believe that this minor scheduling alteration would have a significant impact on the study outcomes.

Individuals were not tested for the presence of HPV DNA at entry as has been the norm for vaccine studies targeting older women. Given the young age at enrolment and HPV DNA prevalence and seroprevalence data for the UK [33]–[35], the risk of prior HPV exposure was thought to be very low. Only a few sera from individuals at enrolment gave positive responses in the neutralization assay. This most likely resulted from a small number of natural infections but may also highlight the limits of assay specificity for some of these pseudoviruses. There was one exception. The proportion of individuals with responses against HPV52 was markedly higher, although the resulting titers tended to be low. A high proportion of antibody positive responses against this pseudovirus in unvaccinated girls and women has previously been reported by ourselves and others [8], [10]. Given the relatively low prevalence of HPV52 infection in the UK [35], [36], this is likely to be a technical issue with the HPV52 pseudovirus itself. Thus, the interpretation of neutralizing antibody responses measured using the HPV52 pseudovirus should be treated with some caution. This is not the case for the other pseudoviruses, however, which demonstrated an apparent assay specificity of around 99-100% in keeping with our previous report [8].

Both vaccines induced high-titer neutralizing antibodies against the vaccine types, HPV16 and HPV18, with responses against HPV16 typically higher than those against HPV18. This appears to be a common finding for both vaccines [8]–[10], [12], [32], [37], [38]. Vaccine type responses were higher in the Cervarix® study arm compared to the Gardasil® study arm up to and including 12 months post first vaccination.

In contrast to the HPV-010 study [39], we found serum neutralizing antibody titers against HPV31 and HPV45 to be higher in the Cervarix® than Gardasil® vaccinees. This discrepancy may be related to the age of vaccination. In the HPV-010 study, the youngest cohort of vaccinees was 18–26 years old and although this group did appear to show some separation of responses between Cervarix® and Gardasil® vaccinees, relative to the older cohorts, these differences were not significant. In our study, participants were 12–15 years old and an age-related improvement in immunogenicity at least for the HPV vaccine Cervarix® has been reported elsewhere [40].

The numbers of individuals seropositive for non-vaccine types HPV31 and HPV45 were lower and their respective neutralization titers substantially lower after two vaccine doses compared to three vaccine doses. This is notably different to the responses generated against the vaccine types for which two doses appear sufficient to elicit high antibody titers [41], [42]. These data suggest that some optimization of a two dose vaccine schedule may be necessary if the generation of cross-neutralizing antibodies against non-vaccine types is considered a desirable outcome of HPV vaccination.

Significant cross-neutralizing antibody responses against other non-vaccine HPV types were fairly common at the peak time point following three doses of vaccine, but restricted to HPV33, HPV35, HPV52 and possibly HPV39 (Cervarix® only) and HPV58 (Gardasil® only). The number of non-vaccine types recognized by vaccinee sera was, in part, related to the magnitude of the responses against the vaccine types, such that the broadest responses were typically seen in the samples that were in the highest third of vaccine type responders.

The majority of Cervarix® and Gardasil® vaccinees tested had detectable neutralizing and binding antibodies against the vaccine types, HPV16 and HPV18, in their genital samples. The titers of serum antibody responses against non-vaccine types were consistently <1% of the titers seen against the vaccine types [8], [10], [39]. Although it is difficult to see how such low levels of cross-neutralizing antibodies in the serum could result in sufficient levels at the site of infection to be protective, our data do at least indicate that such antibodies against HPV31 and HPV45 are present in genital secretions. That vaccine and non-vaccine type neutralizing and binding antibodies can be detected similarly in genital samples and serum, when normalized by IgG content, appears to corroborate the notion that the majority, if not the entirety, of HPV-specific antibodies detected in the mucosa are antibodies that originate from the peripheral blood [12], [17], [32], [43], [44].

Two recent studies using the cervico-vaginal challenge (CVC) model [45] may shed some light on whether such low levels of vaccine-induced antibodies could feasibly be protective at the site of infection. These studies showed that immunization with HPV16 L1 VLP can provide some degree of protection against infection by HPV31, but not HPV58, pseudovirions [46] and passive immunization of HPV16-specific antibodies orders of magnitude lower than those detectable in *in vitro* neutralization assays can provide protection [47]. Taken together these observations suggest that the low levels of cross-neutralizing antibody against some non-vaccine types following immunization with Cervarix® and Gardasil® shown here may be sufficient to confer protection against those types at the site of infection, complicated by the apparently low resolution of the current generation of assays used to detect them.

Finally, there was a strong association between the cross-neutralizing antibody seropositivity reported here and available HPV vaccine trial efficacy data against non-vaccine types. Whether this association reflects a causal role for cross-neutralizing antibodies in the vaccine-induced protection against infection and disease, or an association with the immune effector, or neither, can not be deduced from these data alone. The striking association, however, suggests that concurrent follow up of neutralizing antibody seroprevalence and infection rates in post-immunization populations should be of interest. Thus, while these observations do not, in themselves, establish cross-neutralizing antibodies as correlates of cross-protection they suggest such antibodies may be used as surrogates [48].

These data demonstrate for the first time that cross-neutralizing antibodies can be detected at the genital site of infection and support the possibility that cross-neutralizing antibodies play a role in the cross-protection against HPV infection and disease that has been reported for the current HPV vaccines.

## Supporting Information

Table S1 Safety details.
(DOCX)

Figure S1 **Representative serum swab antibody plots.** **Neutralizing and VLP binding antibody concordance between serum and genital samples.** Neutralization (left panels) and VLP binding (right panels) profiles for two representative Cervarix (A, B) and Gardasil (C, D) vaccinees. Serum (closed circles) and genital (open circles) samples against HPV16 (blue), HPV18 (red) and HPV31 (green).
(TIF)

Protocol S1 Trial Protocol.
(PDF)

Checklist S1 CONSORT Checklist.
(DOC)
