# Supplementary material for: A Randomized, Observer-Blinded Immunogenicity Trial of Cervarix® and Gardasil® Human Papillomavirus Vaccines in 12-15 Year Old Girls
Source: PLoS One. 2013 May 1;8(5):e61825. doi: 10.1371/journal.pone.0061825 (PMC3641072; doi:10.1371/journal.pone.0061825)
Supplement: Table S1 — Safety details. (DOCX) [file pone.0061825.s001.docx]

| **Table S1**. Percentage of participants reporting any indicated symptom | | | | |
| --- | --- | --- | --- | --- |
|  |  | Percentage (95% CI) of participants *^a^* | |  |
| Symptom | Level *^b^* | Cervarix® (n=96) | Gardasil® (n=102) | *p* value *^c^* |
|  |  |  |  |  |
| Loss of appetite | Any | 35.4 (25.9 - 45.8) | 34.3 (25.2 - 44.4) | NS |
|  | Moderate / Severe | 8.3 (3.7 - 15.8) | 8.8 (4.1 - 16.1) | NS |
|  |  |  |  |  |
| Fatigue | Any | 56.3 (45.7 - 66.4) | 50.0 (39.9 - 60.1) | NS |
|  | Moderate / Severe | 19.8 (12.4 - 29.2) | 13.7 (7.7 - 22.0) | NS |
|  |  |  |  |  |
| Headache | Any | 49.0 (38.6 - 59.4) | 47.1 (37.1 - 57.2) | NS |
|  | Moderate / Severe | 20.8 (13.2 - 30.3) | 11.8 (6.2 - 19.6) | NS |
|  |  |  |  |  |
| Fever | ≥37.5°C | 18.8 ( 11.5 - 28.0) | 23.5 (15.7 - 33.0) | NS |
|  | ≥39°C | 0.0 (0.0 - 3.7) | 1.0 (0.0 - 5.3) | NS |
|  |  |  |  |  |
| Redness | Any | 41.7 (31.7 - 52.2) | 38.2 (28.8 - 48.4) | NS |
|  | ≥50mm | 0.0 (0.0 - 3.7) | 1.0 (0.0 - 5.3) | NS |
|  |  |  |  |  |
| Swelling | Any | 25.0 (16.7 - 34.9) | 24.5 (16.5 - 34.0) | NS |
|  | ≥50mm | 3.1 (0.6 - 8.9) | 2.0 (0.2 - 6.9) | NS |
|  |  |  |  |  |
| Pain | Any | 93.8 (86.9 - 97.7) | 86.3 (78.0 - 92.3) | NS |
|  | Moderate / Severe | 24.0 (15.8 - 33.7) | 6.9 ( 2.8 - 13.6) | 0.001 |
|  |  |  |  |  |
| *^a^* Percentage reporting any symptom following any vaccination dose  *^b^* Definitions of moderate/severe symptoms include missing at least 1-2 meals (loss of appetite), sleeping more than usual (fatigue), moderate/severe headache, pain when arm is resting to unwillingness to use arm.  *^c^* Fisher’s exact test. NS, *p*>0.05. | | | | |
