# Supplementary figures and images for: A Randomized, Observer-Blinded Immunogenicity Trial of Cervarix® and Gardasil® Human Papillomavirus Vaccines in 12-15 Year Old Girls
Source: PLoS One. 2013 May 1;8(5):e61825. doi: 10.1371/journal.pone.0061825 (PMC3641072; doi:10.1371/journal.pone.0061825)

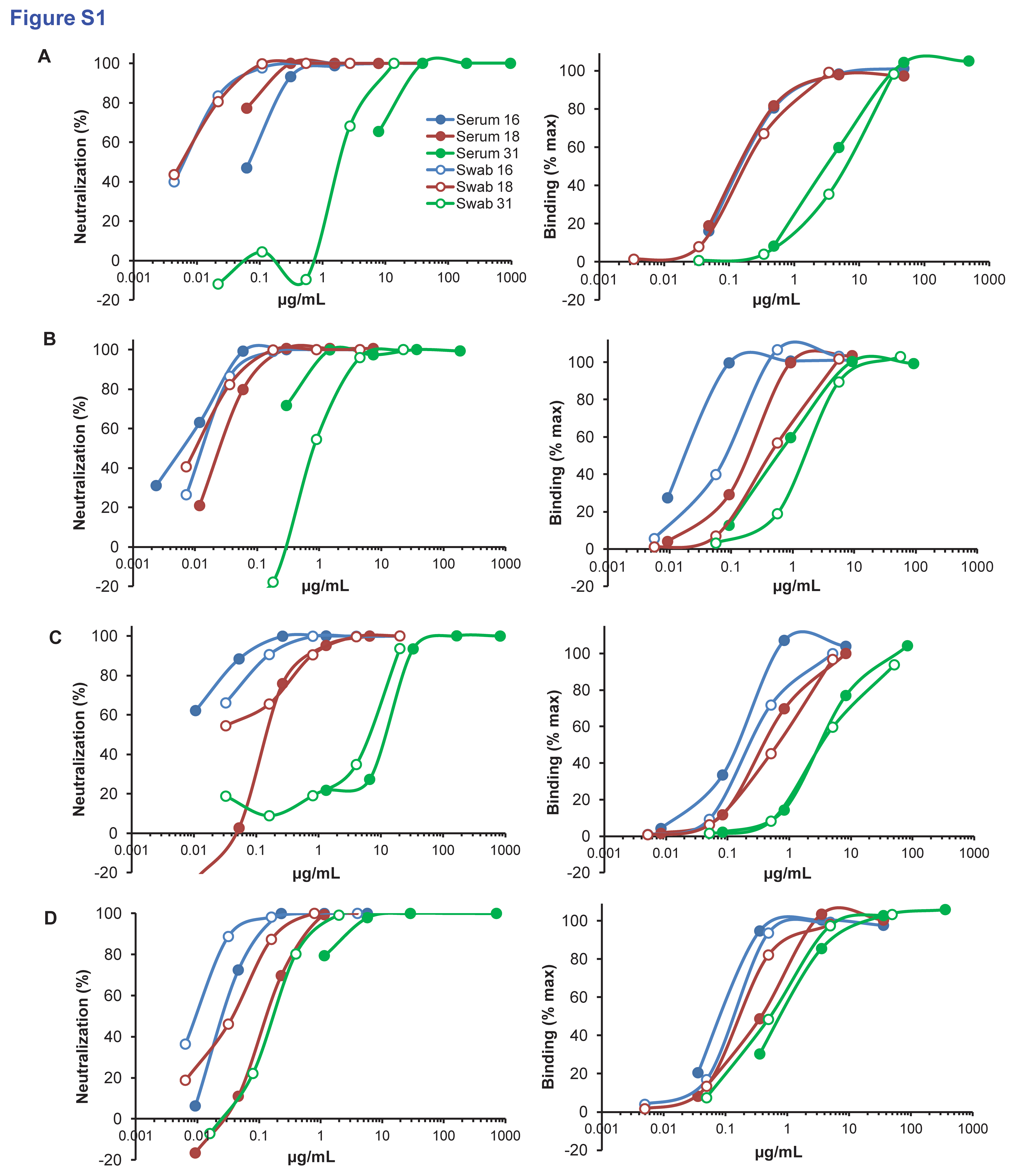

Supplement: Figure S1 — Representative serum swab antibody plots. Neutralizing and VLP binding antibody concordance between serum and genital samples. Neutralization (left panels) and VLP binding (right panels) profiles for two representative Cervarix (A, B) and Gardasil (C, D) vaccinees. Serum (closed circles) and genital (open circles) samples against HPV16 (blue), HPV18 (red) and HPV31 (green). (TIF) [file pone.0061825.s002.tif]
