# Supplementary material for: A Randomized, Observer-Blinded Immunogenicity Trial of Cervarix® and Gardasil® Human Papillomavirus Vaccines in 12-15 Year Old Girls
Source: PLoS One. 2013 May 1;8(5):e61825. doi: 10.1371/journal.pone.0061825 (PMC3641072; doi:10.1371/journal.pone.0061825)
Supplement: Protocol S1 — Trial Protocol. (PDF) [file pone.0061825.s003.pdf]

A Phase IV, randomised study to evaluate the immune responses of UK adolescent girls receiving Cervarix™ or Gardasil™ Human Papillomavirus vaccines (Code: HPV CSP01)

To be carried out by the

NATIONAL VACCINE EVALUATION CONSORTIUM (NVEC)

Incorporating:

CENTRE FOR INFECTIONS, HEALTH PROTECTION AGENCY, LONDON
NATIONAL INSTITUTE FOR BIOLOGICAL STANDARDS AND CONTROL

1. BACKGROUND AND RATIONALE

Cervical screening (the ‘smear test’) helps prevent cervical cancer by finding early signs of abnormalities that can be addressed before they progress to cancer. Another way to prevent disease is to block infection by the human papillomavirus (HPV) types that can lead, usually after many years, to the development of cervical cancer. There are two vaccines now approved for use in many countries worldwide, including the UK: Cervarix™ (GlaxoSmithKline; GSK) and Gardasil™ (Merck).

The UK introduced HPV immunisation for girls in September 2008 using the HPV vaccine Cervarix™. The routine programme will target 12-13 year old girls and there will be a catch-up programme for girls up to 18 years. The first cohort to be immunised are girls born between 1st September 1995 and 31st August 1996 (current school year 8). Immunisation consists of a three-dose schedule over a six month period, starting in Autumn 2008. A schools-based programme has been recommended. The catch-up programme will target all girls born between 1st September 1990 and 31st August 1991 (current school year 13) alongside the first routine cohort (starting in Autumn 2008). The remaining catch-up years will be offered HPV vaccine from September 2009. The Joint Committee for Vaccination and Immunisation (JCVI) advised that a catch-up campaign for all women aged 18 years and over was not cost effective, but that HPV immunisation may be of benefit to individual women and further advice regarding this is anticipated from the Department of Health.

There are many types of HPV virus, some of which are cancer-causing (oncogenic). Both the vaccines (Cervarix™ or Gardasil™) should protect women against two of the most common oncogenic HPV types, 16 and 18, which account for about 70% of cervical cancer. However, as there are many other HPV types that also cause cervical cancer it will be necessary to maintain the cervical cancer screening programme. This is a significant recurrent investment by the NHS to protect the UK population from this devastating disease.

In this study, we will examine the immune responses to immunisation with each vaccine and assess how effective these vaccines may be against other oncogenic types of HPV; this is known as cross-protection. We will also determine if differences in vaccine components and/or their formulation impact on this level of cross-protection. This information will be used to help predict what will happen to each HPV type when the population is vaccinated and so predict gaps in protection that can be filled by the next generation of vaccines. This information may also help to target limited NHS resources to those individuals who may not be sufficiently protected by vaccination.

1.1 Vaccine Characteristics and Immunization Schedules

Cervarix™ and Gardasil™ vaccines contain virus-like particles (VLP) based on the oncogenic HPV types 16 and 18. VLPs are non-infectious and comprise self-assembled L1 (major capsid) proteins. They do not contain the virus genome and so only physically resemble virus particles. Gardasil™ also includes VLPs based on the non-oncogenic HPV types 6 and 11, which are associated with genital warts. There are other differences between the vaccines, summarized in Table 1.1:

Table 1.1 Vaccine characteristics

Characteristic	Cervarix™	Gardasil™
Manufacturer	GSK	Merck / Sanofi Pasteur MSD
VLP	16/18	6/11/16/18
Production source	Insect cells, *Trichnoplusia ni*	Yeast cells, *Saccharomyces cerevisiae*
Dose	20/20 µg per dose	20/40/40/20 µg per dose
Adjuvant	Proprietary ASO4 comprised of 500 µg of aluminum hydroxide and 50 µg of 3-deacylated monophosphoryl lipid A (MPL)	225 µg Aluminum (as amorphous aluminum hydroxyphosphate sulphate)
Immunization	0.5mL IM	0.5mL IM
Recommended schedule	0, 1, 6 months	0, 2, 6 months
Schedule flexibility	None stated	Three doses in 12 month period, minimum 1 month between dose 1 & 2, minimum 3 months between dose 2 & 3

1.2 Available data

Following many small-scale trials examining proof-of-principle issues, several large, multicentre and in many cases, international-scale Phase III vaccine trials have been conducted in young women showing almost complete protection from infection with HPV types 16 or 18 and subsequent occurrence of pre-cancerous (Cervical Intraepithelial Neoplasia, CIN2+) lesions. A few recently published examples are highlighted below.

FUTURE I and FUTURE II were 3 year randomized, double-blind, placebo-controlled studies using the Gardasil™ vaccine. FUTURE I included 5455 healthy women aged 16-24 years old and demonstrated 100% (95% CI = 94-100) protection against cervical lesions associated with vaccine types HPV 16 and 18 (Garland et al., 2007). FUTURE II, included 12,167 health women 15-26 years old and demonstrated 98% (95% CI = 86-100) protection against cervical lesions associated with vaccine types HPV 16 and 18 (FUTURE II Study Group 2007).

The PATRICIA study, a 2 year, randomized, double-blind study using Cervarix™ and Hepatitis A as a control arm was conducted in 18,644 healthy women aged 15-25 years old. This study included participants from Europe, North America, Latin and South America and the Asian-Pacific region. Vaccine-induced protection against high grade CIN2+ lesions associated with the vaccine types 16 and 18 was 90% (95% CI = 53-99) (Paavonen et al., 2007).

As the majority (>95%) of cervical cancers are associated with HPV types belonging to just two distinct Alpha-papillomavirus clades, A9 (16, 31, 33, 35, 52 and 58) and A7 (18, 39, 45, 59 and 68), the extent to which immune responses induced by vaccination with HPV types 16 and 18 will cross-protect with related HPV types is a critical question in the field today. Emerging trial data suggests that some degree of cross-protection is induced by the current HPV vaccines, in particular against HPV types 45 and 31. In the PATRICIA (Cervarix™) study, for example, potential cross-protection against HPV 31 (36·1% [95% CI = 0·5-59·5; p=0·0173]),

HPV 45 (59·9% [95% CI = 2·6-85·2; p=0·0165]) and HPV 52 (32% [95% CI = 3·5-51·9; p=0·0093]) associated cervical lesions. Preliminary data from a Gardasil™ efficacy study demonstrated vaccine efficacy against CIN1-3 or AIS associated with HPV 31 or 45 of 62% (95% CI = 10-85) and associated with HPV 31, 33, 45, 52, or 58 of 43% (95% CI = 7-66) (Brown et al., 2007). A comparison study of Cervarix™ versus Gardasil™ in women between 18 and 45 years of age has recently been initiated and, as of Oct 9th 2008 had completed recruitment, but no data are yet available (NCT00423046: http://clinicaltrials.gov).

This supports the scientific evidence that immunisation of mice with HPV VLPs can induce a limited cross-reactive response against related HPV genotypes; for example HPV 16 VLP induces antibodies against HPV 16 and 31 while immunisation of mice or humans with HPV 18 VLP often induces antibodies against HPV 45 (Giroglou et al., 2001; Combita et al., 2002; Smith et al., 2007).

The mechanism by which these antibodies protect against HPV infection or incident disease is not entirely clear, but is likely to involve antibodies acting to protect the barrier surface of the cervical mucosa. These antibodies could be mucosally-secreted antibodies or peripheral antibodies acting at the cervical site following microtrauma, the predicted mode of infection for HPV. A recent study looked at mucosal antibodies and found a good correlation between HPV antibody levels in the blood and mucosa secretions, although the specificities of these antibodies were not fully described highlighting further need for study in this area (Kemp et al., 2008). The role of HPV-specific T cells in the co-ordination of the immune response against HPV has been examined in some detail over recent years and there is emerging evidence for the generation of limited cross-reactive T cell responses induced by HPV vaccination (e.g. Pinto et al., 2006).

Although the full extent of cross-protection will only become clear over time as incident infections accumulate in both the vaccine and control arms of these multinational vaccine studies, it is certain that the current generation of vaccines will not be adequately protective against all oncogenic HPV types. Thus, an assessment of the limitations of the current generation of HPV vaccines and data helping to inform the formulation of the next generation of HPV vaccines is particularly needed.

2. OBJECTIVES

To undertake a comprehensive comparative evaluation of the HPV-specific immune responses against oncogenic HPV types induced when adolescent female subjects are vaccinated with either Cervarix™ or Gardasil™, by

(i) examining the levels of antibody-mediated cross-neutralisation elicited to oncogenic HPV types following vaccination with either Cervarix™ or Gardasil™

(ii) comparing the levels and patterns of cross-neutralisation induced by each vaccine to highlight potential differences in immunogenicity between the vaccines

(iii) assessing whether there are differences in the specificity of mucosal antibodies or cell-mediated immune responses elicited by each vaccine

3. STUDY DESIGN

This will be a phase IV randomised study comparing the immune responses elicited in young girls (<18 years old) receiving the HPV vaccine Gardasil™ or Cervarix™. Subjects will receive three vaccinations and have four sets of samples collected according to the table below.

**Activity**	**Month**												
Vaccination (Cervarix™ or Gardasil™)	0	1	2	3	4	5	6	7	8	9	10	11	12
Blood taken by Vaccine Research Nurses	0	1	2	3	4	5	6	7	8	9	10	11	12
Voluntary self-taken lower vaginal sponge	0	1	2	3	4	5	6	7	8	9	10	11	12

To facilitate a comparison of immune responses elicited to the two vaccines, the schedules have been synchronized wherein each individual will be vaccinated at months 0, 1 and 6. Schedule flexibility has been highlighted for Gardasil™: Subjects evaluated in the Per Protocol population received all 3 vaccinations within 1 year of enrolment. An analysis of immune response data suggests that flexibility of ±1 month for Dose 2 (i.e., Month 1 to Month 3 in the vaccination regimen) and flexibility of ±2 months for Dose 3 (i.e., Month 4 to Month 8 in the vaccination regimen) do not impact the immune responses to Gardasil™ (www.merck.com/product/usa/pi_circulars/g/gardasil/gardasil_pi.pdf; Merck & Co., Inc. September 2008: #9883608). Calculation of subsequent appointments will be based on the last appointment date rather than time zero.

	**Timepoint (months)**					
**GROUP**	**0**	**1 (-2 days/ +2 weeks)**	**2 (± 1 week)**	**6 (± 1 week)**	**7 (± 1 week)**	**12 (± 1 week)**
**1** (n=200)	Cervarix™ **Blood sample**	Cervarix™	**Blood sample**	Cervarix™	**Samples (incl. extra blood and optional sponge)**	**Blood sample**
**2** (n=200)	Gardasil™ **Blood sample**	Gardasil™	**Blood sample**	Gardasil™	**Samples (incl. extra blood and optional sponge)**	**Blood sample**

"Samples" denotes the collection of:

- 5mL blood (clotted) at Month 0, 2, 7 and 12
- 20-30mL blood (heparinised) at Month 7 may also be taken
- (Optional) self-collected lower vaginal sponge on Month 7

SUBJECT ELIGIBILITY

Inclusion criteria:

- Aged between 12 and 15 years at the time of the first immunisation
- Female
- No contraindications to vaccination as specified in the "Green Book" – Immunisation Against Infectious Disease, HMSO.
- Written informed consent obtained from parent or guardian of subject

Exclusion criteria

- Pregnant or become pregnant during the course of the trial (no contraindications to vaccination for those taking the contraceptive pill).
- Breast-feeding mothers
- Allergic to vaccine components

Deferral

- Subjects who have a fever (axillary temperature ≥ 38℃), or have an acute infection will have injection delayed until resolution
- Not recommended in patients undergoing immunosuppressive therapy
- Not recommended for persons already undergoing vaccination as effect of co-immunisation with other vaccines has not been rigorously tested
- The JCVI (17 Oct 2007, 18 Jul 2008) advises that HPV vaccines and the Td/IPV school leaver booster (low dose diphtheria, tetanus and inactivated polio: Td/IVP) can be co-administered

Withdrawal criteria

- Subject becomes pregnant
- Subject or parent/guardian withdraws consent or lost to follow-up (HPA's SOP on Premature Withdrawal from a Study will be followed).

Girls aged 12 to 15 years will be recruited to this study. It is assumed that the immune response of these girls will not differ from that of girls vaccinated age 12-13 years as part of the routine HPV vaccination.

NUMBER OF SUBJECTS AND DURATION OF STUDY

A total of four hundred (400) subjects will be recruited through two centres: *North Hertfordshire* and *Gloucester*.

It is hoped that recruitment to the study will be completed in a 10-month timeframe. However, as with all adolescent studies, the recruitment rate depends on patient attitude and is largely unpredictable. Experience with previous studies in this age group with the number of Vaccine Research Nurses (VRNs) who will recruit to this study gives a recruitment estimate of 10-15 adolescents per week.

CONDUCT OF THE STUDY

A. Recruitment

Recruitment will be conducted through schools and general practitioners.

General practitioners will be approached to agree to the participation of their surgery in the study and will be asked to document their agreement to the study being conducted in their surgery by the completion of a protocol approval form (Appendix 6). No subjects will be recruited from a surgery until this has been obtained. A site file will be provided to each participating surgery, with documents as listed in the index (Appendix 4). Study investigators and co-ordinators will be available to contact should health professionals or parents require any further information.

Subjects will be recruited from general practices in Hertfordshire and Gloucestershire. Recruitment and follow up will be by dedicated Vaccine Research Nurses (VRNs) currently employed by the Health Protection Agency.

For school-based recruitment, school staff will identify potential participants based solely on age and gender and give/send the initial invitation to participate both to the girl and a parent/guardian. Only girls returning forms requesting further information about the study will be contacted by the Vaccine Research Nurses and contact numbers will be provided for parents and students who require further information about the study. As well as the approved study information leaflet families will be provided with the standard Department of Health information about HPV vaccination and will have the opportunity to discuss the study with dedicated Vaccine Research Nurses and / or medical staff should they wish to do so. Information will be provided in a timely manner to ensure parents/participants have time to seek the opinion of others, should they wish, such as family, friends and other health professionals. Schools will decide the most appropriate methods for identifying potential participants from their registered pupils. It is anticipated that this will almost invariably be via a computerised search of registered female pupils of eligible age.

Written information will be provided about the study and clinicians will discuss the study with individuals and answer any questions before obtaining written informed consent for participation for those who wish to enrol. Because subjects will be under 16 years of age, the *patient information leaflet* will encourage informed discussion of the study with a parent or guardian.

Following confirmation of inclusion and exclusion criteria, subjects will be enrolled in the study and will be allocated to one of the two treatment groups by a randomisation procedure – see below. No study procedure will be undertaken until written informed consent is given by a parent or guardian and the inclusion/exclusion criteria are verified.

GPs will be informed of the participation of each subject in the study by a letter (please see Appendix 1). Provision of this information forms part of the consent procedure.

B. Presentation and Labelling of Study Vaccines
The vaccines to be used in this study are licensed for use in the UK. The study will be registered for Clinical Trial Authorisation (CTA) by the HPA.

Supplies of the vaccines: Cervarix^{TM} and Gardasil^{TM} will be reserved by the CfI from the Department of Health for use in this study. Vaccines will be labelled for the study at HPA CfI according to the appropriate Standard Operating Procedure. The full information required by annex 13 of the GMP rules and guidance is listed below (a-k):

a. name of the sponsor
b. pharmaceutical dosage form, route of administration, quantity of dosage units
c. the batch/code number to identify contents
d. trial subject identification number where applicable
e. directions for use
f. 'for clinical trial use only'
g. the name of the investigator (if not already included as part of a code)
h. a trial reference code allowing identification of the trial and investigator
i. the storage conditions
j. the period of use (use-by date/ expiry date or retest date) in month/year
k. 'keep out of reach of children' except when the product is for use only in hospital

On receipt of the vaccine each dose will have information pertaining to batch, storage conditions, directions for use, expiry date and dosage already printed. HPA will add the label below to the prefilled syringe in order to comply with the full information required by annex 13 of GMP. The HPA's label will contain a statement that the vaccine is for trial-use only, name of Chief Investigator and protocol code. Sample label:

For HPA Clinical Trial Use Only
CI: Prof E Miller
Protocol: HPV CSP01

The additional trial labels will be placed in a position so as not to obscure information such as batch number and expiry date as on the label the vaccine is supplied with. A label containing the same information will be attached to the box containing the vaccine. In addition, if any of the information listed in a-k above is not already printed on the box, a label containing this information will also be attached to the box, taking care not to obscure any information already there.

Vaccine reconciliation or dose accountability will be conducted for all study designated vaccine doses according to standard operating procedures in place at the CfI. The batch numbers for all vaccinations will be recorded by Vaccine Research Nurses (VRNs) on administration.

C. Dispatch of Vaccines to Clinics
The vaccines will be dispatched to the GP clinics or designated vaccine storage area from CfI or Gloucester HPA in appropriate packaging with temperature control and monitoring which will indicate whether the vaccine has been stored at temperatures outside the recommended range. The vaccines will be stored in a secure temperature-controlled environment according to usual practice. If vaccines are damaged or have been exposed to temperatures outside the permitted range, replacement doses will be used.

All vials used will be returned to CfI with Case Report Forms (CRFs) for storage. Any vials that have been wasted or have expired by the end of the study will be disposed of according to local practice, or will be returned to CfI for disposal. Accountability forms will be completed.

D. Vaccination Procedure
The Vaccine Research Nurses (VRNs) will administer vaccinations at designated GP surgeries or schools. Before each dose is given, the VRN will take the subject's temperature. Vaccination will be deferred if the temperature measured is >38°C, or if there is acute illness on the day of vaccination.

The VRN will administer vaccines by intramuscular injection in the non-dominant arm. Before each dose is given the VRN will record that the vaccine has been maintained under appropriate storage conditions. The VRN will write the subject's initials and date onto an adhesive label bearing the subject number and stick this onto the empty vaccine containers, which will be returned with the CRFs to CfI.

Standard immunisation practices and appropriate precautions for any anaphylactic reaction will be followed. The HPA's SOP on Reporting Serious Adverse Events (SOP28) will be followed for classifying events and reporting SUSARs to the appropriate authorities and manufacturers.

Participants' GPs will be informed that HPV vaccination has been given after each dose. After the blood sample visit at month 12, GPs will be informed which HPV vaccine the participant received.

E. Randomisation
A computerised block randomisation list will be produced by the Statistician at CfI. Each VRN will be allocated blocks of sequential numbers in accordance with the block size used for randomisation. On recruitment to the study, each subject will be allocated, in order of inclusion, the next available study number by their VRN. The study number will define the group to which the subject is assigned and therefore the vaccine they will receive. Vaccinated individuals, however, will not be aware of which HPV vaccine (Gardasil^{TM} or Cervarix^{TM}) they will be receiving during the course of the study. Laboratory staff will be aware of the study number and patient initials associated with the collected samples but will not be able to link these data with the vaccine received nor other patient identifiers.

F. Collection and testing of samples
Four blood samples (ca. 5mL clotted blood) will be collected from each subject, at months 0, 2, 7 and 12 of the study. One extra blood sample (ca. 30mL heparinised blood) may be collected at month 7 i.e. one month following the final vaccine dose. An optional self-taken lower vaginal sponge will also be collected on month 7. Participation in the study will not depend on whether the individual volunteers to give a sponge sample.

Forms from appropriate Standard Operating Procedures (SOPs) will be included with samples to document the movement of samples between sites and laboratories. Logs of samples sent and received will be kept at CfI to enable the identification of any lost or delayed samples and provide a log of where samples are currently stored. Laboratory staff will be blinded to the vaccine received.

Blood
Blood sampling will be in accordance with venepuncture SOP. No more than two attempts at venepuncture will be made. An anaesthetic cream (EMLA or Ametop) will be offered for use on the intended bleeding site to minimise discomfort. At least 5ml of blood and no more than 10 ml of blood will be taken at each sample collection point for serum generation. At month 7 (one month following the full regimen of Gardasil^{TM} or Cervarix^{TM} doses) an additional 30mls of

heparinised blood may be taken for the isolation and storage of peripheral blood mononuclear cells (PBMC; white blood cells).

The Vaccine Research Nurse (VRN) will stick an adhesive label bearing the subject's unique number onto each sample tube. She will also write the subject's initials on each label. Adhesive labels bearing the subject number will be packed with the samples and sent to the primary receiving laboratory, which will be:

FAO: Dr Simon Beddows [CSP01]
VIA **Central Specimen Reception**
Centre for Infections
Health Protection Agency
61 Colindale Avenue
London NW9 5EQ

VRNs will send standard 5mL blood samples taken at **Months 0**, **2**, and **12** by Royal Mail next day delivery. Blood samples taken at **Month 7** (standard 5mL blood plus 30mL heparinised blood) will be sent by courier for same day or overnight delivery, as appropriate.

Blood will be processed and tested or archived according to laboratory SOPs for measurement of the following:

i. HPV antibodies using binding and functional assays
ii. HPV cell mediated immune responses

(Optional) Lower Vaginal Self-Taken Sponge
A self-taken lower vaginal sponge will be collected at **Month 7** and sent by courier with the **Month 7** blood samples, as above, if applicable. This sample is voluntary and refusal to take part will not affect further participation in the study. Instructions on how to take the sponge will be given by the Vaccine Research Nurse. Essentially, a sterile sponge is inserted 1-2cm into the lower entrance of the vagina, and the sides of the vagina are sponged. The sponge is then placed into transport medium and labelled with an identification sticker. These samples will be tested for HPV-specific antibodies generated to the vaccine.

On completion of testing, samples will be archived at the Health Protection Agency, Colindale, where permission for this has been granted by the subject.

I. Collection of safety data
Subjects will be asked to record daily in a health diary any local reaction (redness, swelling, tenderness) and give measurements if present, to take their temperature using a thermometer provided. They will be asked to record any visits to a doctor – hospital or GP – as well as to document anything else that they think study personnel should be aware of.

H. Data records
Vaccine Research Nurses (VRNs) will maintain their own records of all subjects enrolled in the study under their care. Information kept will include the surgery or school attended, vaccination due dates, contact details.

CRFs will be provided to VRNs by CfI. CRFs will be numbered and will indicate the group to which the subject assigned that number has been randomised. Subjects will not be made aware of this information. CRF packs will comprise a series of cards relating to each clinical stage of the study process. Also included will be a change of circumstances card for any

subject whose name or address is altered during the course of their participation, and a completion card that will detail whether the protocol was completed or whether subjects were withdrawn or lost to follow-up, with appropriate details given.

VRNs will complete the CRFs at each visit and these will be sent to CfI. A study database will be constructed at CfI to record the information collected in the CRFs. As the data is being entered, the CRFs will be monitored for completion errors or omissions. When such a problem is identified the card will be photocopied and the field for correction marked. The photocopy will be sent to the VRN who will make the correction, crossing out any incorrect information with a single line, and will sign and date the change. On return of the photocopy to CfI the database will be updated accordingly and the photocopy filed with the original CRF.

Information from the clinical record cards will be entered at CfI into a database on a microcomputer. Data will be entered twice by two independent data-entry staff and will be verified electronically. Laboratory data will be e-mailed to the Consultant for Clinical Trials and imported into the database so that test results can be linked to clinical records.

STATISTICAL METHODS

Full details will be given in the statistical analysis plan produced by the Statistician.

Brief description.

The primary purpose of this study is to determine the level of neutralising antibody reactivity (i.e. antibodies able to prevent HPV infection of target cells in laboratory culture) against not only the vaccine-incorporated types (HPV 16 and 18) but also the genetically-related, non-vaccine cancer-causing types (31, 33, 35, 39, 45, 52, 58, 59, 68). The main outcome will be the proportion of vaccinated individuals generating measurable antibody-mediated neutralisation against HPV 45 at the peak response (one month post-3rd dose; i.e. month 7). Further outcome measures will be the proportion of vaccinated individuals generating measurable antibody-mediated neutralisation against non-vaccine HR HPV types and the geometric mean titres for each HPV type, by vaccine and sample type, at each time point. For proportions exact confidence intervals will be calculated. For titres data will be logged (base 10) and assuming normality on the log-scale 95% confidence intervals calculated. If for any reason data are clearly not normal then the 95% confidence interval may be calculated after excluding outliers (such as those individuals who fail to generate an acceptable immune response to vaccine-incorporated HPV types, 16 and 18). If a large proportion of the subjects fail to generate an acceptable response then it may not be possible to calculate geometric means.

SAMPLE SIZE CALCULATION

There are limited data regarding cross-reaction to non-vaccine HPV types. Clinical trials have not been powered to give precise estimates of protection against individual non-vaccine types, and where data have been reported these are commonly limited to cross-protection against the two next most common HPV types HPV 31 and HPV 45. Trials have estimated that protection against HPV 31 ranges from ~10% to 55% and against HPV 45 from ~60% to 90%; however, the confidence limits around these estimates are extremely wide (Paavonen et al., 2007; Brown et al., 2007).

The sample size estimates for this study are based on the assumption that the percentage of blood samples at month 7 with cross-reactive antibodies against HPV 31 will be between 10% and 55% and against HPV 45 between 25% and 90% (Giroglou et al., 2001; Combita et al., 2002; Smith et al., 2007). Recruitment of 200 participants in each arm of the study will enable

us to detect the percentage of cross reactive samples at month 7 within the limits shown in the following table.

Cross reaction against:	Percentage of samples showing cross-reaction	Precision of estimate of the percentage of samples showing cross-reaction
HPV 31	10%	+/- ~4%
	55%,	+/- ~7%
HPV 45	25%	+/- ~6%
	90%	+/- ~4%

When comparing the two vaccines, the population percentages which give rise to an 80% or more chance that observed percentages in the two study arms will be found to be significantly different at the 5% level are shown below. For example, we would have an 80% chance of detecting as significantly different, 50% versus 35% cross-reactive antibodies in the two populations.

Percentage of samples with cross-reactive antibodies in **Population 1**	Percentage of samples with cross-reactive antibodies in **Population 2** (percentages that give rise to an 80% or more chance that the observed percentages in the two study arms will be found to be significantly different)	
10%	≤2.7%	≥20.5%
25%	≤13.5%	≥38.5%
50%	≤35.7%	≥64.3%
75%	≤61.5%	≥86.5%
90%	≤79.5%	≥97.3%

DATA ANALYSIS AND PRESENTATION

Data analysis will be undertaken by the Immunisation Department Statistician and members of the Clinical Trial Team where appropriate. The results of the study will be reported to the Department of Health, the Joint Committee on Vaccination and Immunisation and a paper submitted for publication in a peer-reviewed journal. Further details will be in the statistical analysis plan.

COMPLIANCE WITH GUIDELINES

The study will be conducted in accordance with the ICH GCP (1996) guidelines, the Declaration of Helsinki (2008), and the EU Clinical Trial Directive (2004). Approval for the study will be sought from the appropriate research ethics committee(s). The HPA will obtain a Eudract registration number for the trial. The HPA will then apply for CTA status for the study from the Medicines and Health Care Products Agency (MHRA).

LABORATORY TESTING OF VACCINES

All batches marketed in the UK and in routine use at the time of the study will have been released by EC Official Control Authority Batch Release process and tested according to documented guidelines by an Official Medicines Control Laboratory. The National Institute for Biological Standards and Control (NIBSC) or the National Institute for Public Health and the Environment (RIVM; The Netherlands) will have tested and reviewed the summary batch protocols for Gardasil™ and NIBSC or the Scientific Institute of Public Health (SIPH; Belgium) will have tested and reviewed the summary batch protocols for Cervarix™. No additional laboratory testing of the vaccines supplied for the study will be necessary.

INDEMNITY ARRANGEMENTS
Product liability will apply as both vaccines are licensed for use in the UK. The Sponsor for this study will be the Health Protection Agency, and indemnity will be provided under the NHS indemnity scheme wherein cover for any negligent harm caused by our staff as a result of study participation and where justified, an *ex gratia* payment for any non-negligent harm, will be provided as appropriate.

STUDY PERSONNEL
Please see Appendix 2

TARGET DATES
Recruitment started in September 2009 and is anticipated to end in autumn 2010.The final dose of the vaccine (Month 6) will be given spring 2010. The trial will last 12 months. The final samples are expected to be collected between autumn 2011.

References

Brown D, et al "HPV Type 6/11/16/18 Vaccine: First Analysis of Cross-Protection against Persistent Infection, Cervical Intraepithelial Neoplasia (CIN), and Adenocarcinoma In Situ (AIS) Caused by Oncogenic HPV Types in Addition to 16/18." Abstract G-1720b: 47th Interscience Conference on Antimicrobial Agents and Chemotherapy, Sept.17-20, 2007

Combita AL, Touze A, Bousarghin L, Christensen ND, Coursaget P. Identification of two cross-neutralizing linear epitopes within the L1 major capsid protein of human papillomaviruses. J Virol 2002; 76: 6480–86.

Department of Health (UK) Announcement: 02 May 2008 - INTRODUCTION OF HUMAN PAPILLOMAVIRUS VACCINE INTO THE NATIONAL IMMUNISATION PROGRAMME

Department of Health (UK) Announcement: 19 June 2008 - INTRODUCTION OF HUMAN PAPILLOMAVIRUS VACCINE INTO THE NATIONAL IMMUNISATION PROGRAMME: announcement of vaccine to be used

FUTURE II Study Group. Quadrivalent vaccine against human papillomavirus to prevent high-grade cervical lesions. N Engl J Med 2007;356:1915–27.

Garland SM, Hernandez-Avila M, Wheeler CM, Perez G, Harper DM, Leodolter S, et al. Females United to Unilaterally Reduce Endo/Ectocervical Disease (FUTURE) I Investigators. Quadrivalent vaccine against human papillomavirus to prevent anogenital diseases. N Engl J Med 2007;356:1928–43.

Giannini SL, Hanon E, Moris P, Van Mechelen M, Morel S, Dessy F, et al. Enhanced humoral and memory B cellular immunity using HPV16/18 L1 VLP vaccine formulated with the MPL/aluminum salt combination (AS04) compared to aluminium salt only. Vaccine 2006;24:5937–49.

Giroglou T, Sapp M, Lane C, et al. Immunological analyses of human papillomavirus capsids. Vaccine 2001; 19: 1783–93.

Kemp TJ, García-Piñeres A, Falk RT, Poncelet S, Dessy F, Giannini SL, Rodriguez AC, Porras C, Herrero R, Hildesheim A, Pinto LA; for the Costa Rica Vaccine Trial (CVT) Group.Evaluation of systemic and mucosal anti-HPV16 and anti-HPV18 antibody responses from vaccinated women. Vaccine. 2008 Jul 4;26(29-30):3608-3616. Epub 2008 May 19.

Paavonen J, Jenkins D, Bosch FX, Naud P, Salmerón J, Wheeler CM, et al. HPV PATRICIA study group. Efficacy of a prophylactic adjuvanted bivalent L1 virus-like-particle vaccine against infection with human papillomavirus types 16 and 18 in young women: an interim analysis of a phase III double-blind, randomised controlled trial. Lancet 2007;369: 2161–70.

Pinto LA, Viscidi R, Harro CD, Kemp TJ, García-Piñeres AJ, Trivett M, et al. Cellular immune responses to HPV-18, -31, and -53 in healthy volunteers immunized with recombinant HPV-16 L1 virus like particles. Virology 2006;353:451–62.

Smith JF, Brownlow M, Brown M, Kowalski R, Esser MT, Ruiz W, et al. Antibodies from women immunized with Gardasil ((R)) cross-neutralize HPV 45 pseudovirions. Hum Vaccin 2007;3:109–16.

Appendix 1:

Patient Information:
Pre-information leaflet, Full information leaflet, Consent form, Introduction letter, GP information letter

HPA headed paper

HPV Vaccine Study

- Your GP / School [will insert in final letter as appropriate] is working with us to carry out a study of two licensed vaccines against HPV, which is the virus that can cause cervical cancer

- We would like to invite you to take part in this study as you are a female **aged between 12 and 15 years**

- We would like your permission for a Vaccine Research Nurse to contact you for an informal discussion and to give you further information about this study.

- Please note that we have not been given your name and address details.

➢ **If you would like further information please complete the slip at the bottom of this leaflet and return in the envelope provided.**

➢ **If you do not want a Vaccine Research Nurse to contact you, you need do nothing further. This will not affect your routine medical care.**

Thank you

✂--

I would like to receive further information about this study and I am happy for a Vaccine Research Nurse to contact me.

Name: ____

Address:____

____

Tel No:__________________**Mobile no.**_________________

Preferred time to be contacted:___________________________

GP Surgery I attend: _________________________________

School I attend [to be inserted if appropriate]: ________________

(HPV CSP01 study)

GP / School covering letter for pre information leaflet

ON SURGERY / SCHOOL HEADED PAPER

Dear Patient / Student,

We are working with the Health Protection Agency* on a study of two HPV vaccines. HPV is the virus that has been shown to cause cervical cancer.

As you are a female aged between 12 and 15 years, we are sending you this letter and a leaflet that gives a brief summary of the study and how to receive more information.

Please note that the HPA does not have your name or address details and will only contact you if you request further information.

Yours sincerely

Signature & name [GP or School representative as appropriate]

*The Health Protection Agency (HPA) is an independent body funded by the government that protects the health and well-being of the population. The Agency plays a critical role in protecting people from infectious diseases and in preventing harm when hazards involving chemicals, poisons or radiation occur.

Patient Information Leaflet

HPV Vaccination Study

Full title: A Phase IV, randomised study to evaluate the immune responses of UK adolescent girls receiving Cervarix^{TM} or Gardasil^{TM} Human Papillomavirus vaccines

Study code: HPV CSP01

Your doctors' surgery or school is taking part in a study with the Health Protection Agency of two licensed HPV vaccines, one of which is being used in the routine UK schedule. Before you decide if you would like to join this study you need to understand the reasons the research is being done and what it would involve. Ask us if there is anything that is not clear or if you would like more information. We encourage you to discuss this leaflet with your parents and others to help you with your decision. It is important to take time to decide whether or not you wish to take part.

Part 1 *tells you the purpose of the study and what it would involve*

Part 2 *gives you more detailed information about the conduct of the study*

PART 1

What is the purpose of the study?
HPV is a virus that can cause cervical cancer in some women. There are many strains of HPV. About 12 HPV strains can cause cervical cancer, two strains of HPV can cause genital warts and many other HPV strains cause no disease. Around 70% of cervical cancers are caused by just two strains (known as HPV 16 and HPV 18). Many of the other strains that can cause cervical cancer are very similar to these two strains.

Two vaccines against HPV, called Cervarix^{TM} and Gardasil^{TM}, have recently been licensed in the UK. These vaccines protect against infection with HPV 16 and HPV 18 (the strains that cause 70% of cervical cancers). Gardasil^{TM} also protects against the two strains (HPV 6 and HPV 11) that can cause genital warts. The study will assess both vaccines (Cervarix^{TM} and Gardasil^{TM}).

We are interested in whether the HPV vaccines will protect against infection with other strains similar to those included in the vaccine. To do this, we will look at the antibodies produced by your body after vaccination and use these in laboratory experiments to see which strains of virus they work against. Antibodies are the substances in your blood that help you fight infections. Some antibodies, called mucosal antibodies, are found on the outside of the body (such as the genitals and mouth) and protect against infection at those sites. We will examine white blood cells after the third dose of vaccine: it is these cells which co-ordinate the production of antibodies in the body.

Is HPV vaccine routinely given in the UK?
Since September 2008, HPV vaccine is being given routinely to 12-13 year old girls (school year 8). The vaccine being used is Cervarix^{TM}. Older girls aged up to 18 years will also be offered the vaccine in a catch-up programme. Both HPV vaccines are licensed for use in the UK. If you do not

wish to take part in this study then you will still be able to have the Cervarix™ HPV vaccine in the future. Most people will be offered the vaccine through their school. Your local primary care trust (PCT) will let you know when you are due to be given the vaccine.

How effective are the HPV vaccines?
Both HPV vaccines are 99% effective in preventing pre-cancerous cervical abnormalities caused by the HPV types 16 and 18. Pre-cancerous abnormalities can lead to cervical cancer if left untreated. HPV 16 and 18 cause about 70% of cervical cancers. HPV vaccines work by preventing initial HPV infection. HPV is sexually transmitted and is extremely common: about 40% of 20-25 year old women have HPV infection.

The vaccines have been shown to provide high levels of protection for at least six years. It is likely that protection will last much longer than this however as antibody levels in vaccinated women in clinical trials have remained very high.

For further information please visit: www.immunisation.nhs.uk/Vaccines/HPV

Vaccine Safety
Both HPV vaccines have been shown to be safe in clinical trials and are licensed for use in Europe. In clinical trials alone 60,000 women have received HPV vaccine and HPV vaccination is now recommended for girls in many developed countries.

As with most vaccines, the most common side effect is mild to moderate swelling, redness and pain at the site where the injection is given. Other mild side effects, such as slightly raised temperature, sickness, dizziness, diarrhoea and muscle aches have been reported. Very rarely, as with most vaccines, some people have an allergic reaction soon after immunisation. The Vaccine Research Nurse will know how to treat this.

For further information please visit: www.immunisation.nhs.uk/Vaccines/HPV

Can women take part if they are pregnant?
You will be asked by the Vaccine Research Nurse (VRN) about the possibility that you may be pregnant. If you are pregnant or breast-feeding you cannot take part in this study. This is because HPV vaccination for pregnant women has not been well studied. Whilst you are in this study, if you are having sex, you must use an effective method of contraception to reduce the risk of pregnancy. If you would like, you can discuss this with a nurse/doctor at your GP practice.

Will I benefit from taking part in this study?
You may not benefit from taking part in this study as you may be given the same vaccine as most other girls in the UK (Cervarix™). However you may benefit as you may be given Gardasil™. Gardasil™ is licensed in the UK but is not being used for most girls because it is more expensive. Both vaccines will protect you from infection with two types of HPV that cause 70% of cervical cancers. If you are given Gardasil™ you will also be protected against genital warts. By taking part in this study you will benefit girls and women in the future because the results from this study will help decide future vaccination policy from the Department of Health.

Are there any disadvantages if I take part?
If you take part in this study you will have 4 blood samples taken. Having a blood sample taken may occasionally result in a bruise, which will usually disappear after a couple of days. You will also be asked for an entirely voluntary self-taken lower vaginal sample. You will be able to take this sample in private and it should not cause discomfort. If you do not wish to provide this sample you can still take part in the study.

Do I have to take part?
No. You can choose whether you would like to take part in this study. If you decide to take part after discussion with your VRN, you and your parent/guardian will be asked to sign a consent form. Taking part in this study is entirely voluntary, you can stop at any time without giving a reason, and your VRN will be happy to discuss any further vaccinations that should be given by your GP surgery or at school.

Why have I been invited to take part?
We wish to examine the body's responses to the HPV vaccines Cervarix™ and Gardasil™ to determine the likelihood that the current vaccines will protect against other cancer-causing strains of HPV that are not present in the vaccines. We would like to invite you to take part in a study that will enrol 400 girls in Hertfordshire and Gloucestershire. Girls aged 12 to 15 years at your GP surgery or school will be invited to take part as these girls are closest in age to those who will usually be given the HPV vaccine in the UK.

What will happen to me if I take part?
If you choose to take part you will receive the HPV vaccine and be asked to give 4 blood samples and, if you agree, provide one self-taken lower vaginal sample. The study will involve 6 visits to see the VRN at your GP surgery or school over 12 months (as shown in the table below). The first appointment will take about an hour and the rest about half an hour each. We will inform your GP that you are taking part in the study.

**Activity**	**Month**												
Vaccination (Cervarix™ or Gardasil™)	0	1	2	3	4	5	6	7	8	9	10	11	12
Blood taken by Vaccine Research Nurse	0	1	2	3	4	5	6	7	8	9	10	11	12
Voluntary self-taken lower vaginal sponge	0	1	2	3	4	5	6	7	8	9	10	11	12

1. HPV vaccinations
All people taking part in the study will be given three doses of HPV vaccine at months 0, 1 and 6 of the study (highlighted in yellow in the table, above). All vaccines will be given at your GP surgery or school by your VRN. There are two groups in this study, girls in one group will be given Gardasil™ and girls in the other group will be given Cervarix™. A computer programme will decide randomly (like tossing a coin) which group you are in. This random draw helps us make sure the groups are similar when we are comparing the two HPV vaccines.

2. Blood Samples
Blood samples will be taken at four visits (months 0, 2, 7 and 12 highlighted in blue on the table above). At each visit, your VRN will offer you some cream to numb the skin and will take one tube (5-10mls) of blood. At month 7, a larger tube (30ml) may also be filled with blood. The blood samples will be used to measure blood antibodies and cells and compare them between the two groups.

3. Self-taken lower vaginal sample
We would like you to provide one self-taken lower vaginal sponge at month 7 (highlighted in green on the table above). If you do not wish to provide this sample you can still take part in the study. If you would like to decide nearer to the time (month 7), you can still take part in the study. If you do decide to provide this sample, the VRN will give you the self-taken sample kit and talk to you about the sample in months 6-7. You will be able to take the sample in private and it should not cause any discomfort. This sponge sample will be very useful because it will let us look at the mucosal antibodies. These are the antibodies that are outside the blood at the genital surface and will be the body's first defence against HPV infection.
The consent form includes consent to provide a lower vaginal sponge sample. If you agree to provide this sample please initial the box. If you initial the box and then change your mind and do not wish to provide the sample, please tell the VRN. If you do not initial the box and later change your mind and wish to provide a lower vaginal sponge sample, please talk to your VRN.

4. Health diary and records

We will ask you to complete a health diary to record how well you have felt after each vaccination (e.g. any problems, reactions at injection site, temperature, if you have seen a doctor, etc). Your VRN will explain how to fill in the diary and will show you how to use the thermometer you will be given. We will review your medical notes before each vaccination to collect information about any illnesses or medicines prescribed. We may then contact your doctor for further information. If you have a serious illness and see the GP or are admitted to hospital, please inform the doctor that you are taking part in this study. Regardless of the illness or reason for admission, please then let us know by telephoning your VRN.

The samples collected for this study and the health diary would not be part of your routine care if you do not take part in the study.

At the end of the study, we will inform you of the overall results of the study and which vaccine you received. We will not tell you or anyone else your individual results.

Expenses and payments

If you incur any costs when seeing the VRN (to have the vaccine or give samples) which you wish to claim back, please ask your VRN for a claim form and we will reimburse you.

Will my taking part in the study be kept confidential?

Yes, we will follow ethical and legal practice and all information will be handled in confidence. For further details on confidentiality, see **Part 2** of this information sheet.

What happens when the research study stops?

When the study ends we will tell you which of the HPV vaccines you were given. If you completed the study, you will not need to have the HPV vaccine when you are offered it through the PCT (probably through your school). If you stop taking part in the study early then we will let your GP know as you will need to complete your vaccination under the national HPV immunisation programme.

After we have completed this study, there may be small amounts of your samples remaining. We would like to be able to use these samples for other studies that will help us understand better how vaccines work. Before we do this we will remove your name and any other information from which you could be identified. If you are happy for us to use your left-over samples for other studies, please consent to this on the consent form. If you do not wish your left-over samples to be kept for future studies, you can still take part in this study.

Part 1 summary:

- **You will be given the usual course of three HPV vaccinations, have four sets of blood samples taken plus one optional self-taken lower vaginal sample**
- **The study will take a year and involve 6 visits to the Vaccine Research Nurse – either at your GP or School.**
- **The vaccines we are using are licensed for use in the UK and Europe**

If the information in Part 1 has interested you and you are considering taking part please read the additional information in Part 2 before making any decision.

This is the end of Part 1

PART 2

What if relevant new information becomes available?
If relevant new information becomes available or the study is to be stopped for any reason we will let you know, as well as give you and your GP information about your continuing care if appropriate.

What will happen if I do not want to carry on in the study?
You can withdraw from the study at any time without affecting your routine care. We will let your GP know so you can complete your vaccination under the national HPV immunisation programme.

If you withdraw you would need to decide if you want samples collected from you to be destroyed. If you would like them to be destroyed you would need to inform us in writing. We would retain any information collected to that point in case we needed to reach you at some point in the future based on the outcome of the study.

What if there is a problem?

- **Complaints**

If you have a concern about any aspect of this study, you can contact the study organiser, Prof Elizabeth Miller on 0208 327 7434, who will do her best to answer your questions. If you remain unhappy and wish to complain formally then you can do this through the NHS Complaints Procedure, details of which can be obtained at www.nhs.uk or by phoning 0845 601 3012

- **Harm**

The vaccines that will be given in this study are licensed for use in the UK. In the event that something does go wrong and you are harmed during the research and this is due to someone's negligence then you may have grounds for legal action for compensation against the Health Protection Agency but you may have to pay your legal costs. The National Health Service complaints mechanism will still be available to you. Where justified, an *ex gratia* payment for any non-negligent harm may be made.

Will my participation in the study be kept confidential?
Under the data protection laws, you have a right know how your personal data from this trial will be used so you can understand what information will be collected and who will have access to it. We will collect only sufficient data to enable us to conduct the study and answer the study questions. The people who require data will only be given information about you that is necessary for their task, for example, people analysing samples and study results will not be told your name or address. The only people who may be given access to study information will be employees or agents of the Health Protection Agency, the Department of Health or regulatory authorities who may wish to check the study is being carried out within the appropriate guidelines.

The data will only be used for the purposes of this study and any data released outside the above group will be anonymised. This means that your name and address will be removed. Data will be stored in secure Health Protection Agency facilities and will be disposed of within clinical trial guidelines after the appropriate time period which may be a number of years.

Will my GP be involved in this study?
We will write to your GP to let him/her know that you are taking part in the study. During the study, your GP will not be involved in giving the vaccine or taking samples – the VRN will do these tasks.

What will happen to any samples I give?
The samples will be labelled with your study number and initials but not your name. The samples will be tested in laboratories and results sent to the Health Protection Agency, Centre for Infections where they will be linked with information about you.

Will any genetic tests be done?
No genetic tests will be carried out on your samples.

What will happen to the results of the research study?
We plan to publish the results in a medical journal which will be accessible to the public. The results of the study will also be reported to the Department of Health to help with planning national vaccination programmes in the future. You will not be named in any report or publication and it will not be possible for anyone to work out that you took part in the study.

Who is organising and funding the research?
The study is funded by the UK Department of Health and is being organised by the Immunisation Department of the Health Protection Agency. The Health Protection Agency (HPA) is an independent body that protects the health and well-being of the population. The Agency plays a critical role in protecting people from infectious diseases and in preventing harm when hazards involving chemicals, poisons or radiation occur.

Who reviewed the study?
All research in the NHS is looked at by an independent group of people, called a Research Ethics Committee to protect the safety, rights, well being and dignity of individuals. This study has been reviewed and given favourable opinion by the XXXX Research Ethics Committee (Tel No. xxxxxxxxxxx). The Research Ethics Committee reference number for this study is…

Further information and contact details:
If you have any questions, you can ask your VRN or contact the organisers, Professor Elizabeth Miller on 0208 200 6868 or Dr Chee Yung 020 8327 7603.

Further information about our trials programme including links to the organisations reviewing research and the guidance we follow can be found at www.hpa.org.uk and typing *clinical trials* into the search bar.

We do hope that you will take part in this study. Your contribution would be an important step towards the continual improvement of vaccine policy in the UK. You may be invited to take part in future studies with us by virtue of the surgery you attend. We reassure you that invitations for future studies will not be because of anything to be concerned about from results of this study.

Sticker with Vaccine Research Nurse Name and Contact Details

CONSENT FORM

Centre: **Gloucester / Hertfordshire** (delete as appropriate)
Study Number: **HPV CSP01**

Patient Identification Number for this trial:

Title of Project: A Phase IV, randomised study to evaluate the immune responses of UK adolescent girls receiving Cervarix^{TM} or Gardasil^{TM} Human Papillomavirus vaccines

Name of Chief Investigator: **Professor Elizabeth Miller**

	**Patient/Student please initial box**	**Parent/Guardian please initial box**
1. I confirm that I have read and understand the information sheet dated.................... (version............) for the above study. I have had the opportunity to consider the information, ask questions and have had these answered satisfactorily.		
2. I understand that my participation is voluntary and that I am/my daughter is free to withdraw at any time without giving any reason, without my/her medical care or legal rights being affected.		
3. I understand that relevant sections of my/my daughter's medical notes and data collected during the study, may be looked at by individuals from the Health Protection Agency, from regulatory authorities or from the NHS Trust, where it is relevant to my taking part in this research. I give permission for these individuals to have access to my/her records.		
4. I agree to my/my daughter's GP being informed of my/my daughter's participation in the study		
5. I agree to take/my daughter taking part in the above study.		
6. I agree to provide /my daughter providing a self-taken lower vaginal sponge sample. [This is optional – you/she may still take part in the study without providing this sample.]		
7. I consent to the use of any residual samples from me/my daughter being used within the Health Protection Agency, once anonymised, to improve the understanding of vaccines and how they work. [This is optional – you/she can still take part in the study if you do not give consent for this. If you do not wish samples to be stored please strike through this section and do not initial the box].		

Patient / Student (Participant)	Date	Signature
Parent / Guardian of Patient	Date	Signature
Person taking consent	Date	Signature

When completed, 1 copy for participant; 1 copy for researcher site file (and scanned into medical notes) and original to be held at Centre for Infections

ON SURGERY / SCHOOL HEADED PAPER

Dear Patient / Student

I am the Vaccine Research Nurse at your doctor's surgery / school [will insert as appropriate]. Thank you for requesting information about the study of vaccines taking place at the surgery. Please find enclosed a leaflet that gives full details of the study and what would be involved should you wish to enrol.

I will try to contact you by telephone shortly to see if you would like to discuss any aspect of the study or have any queries. Participation in the study is entirely voluntary. If you decide not to participate, it will in no way affect your routine care.

If you would like to take part in the study you can also call me on the telephone numbers listed at the bottom of this page. (If you prefer to decline you need take no action, or your can call me to let me know you would like your name removed form my contact list) I look forward to hearing from you and thank you for your time and consideration.

Yours sincerely

VACCINE RESEARCH NURSE
CONTACT DETAILS

ON HPA HEADED PAPER

HPV vaccine study GP Information Leaflet

A Phase IV, randomised study to evaluate the immune responses of UK adolescent girls receiving Cervarix™ or Gardasil™ Human Papillomavirus vaccines (Study No.: **HPV CSP01**)

Dear Doctor Study No. **HPV CSP01**

Re : ____

A study to evaluate the response to licensed HPV vaccines is being conducted in your health district. This study seeks to assess antibody responses to these vaccines with the schedule below, including assessing any cross-protection that the vaccines may afford against HPV types not included in the vaccines. Schedule flexibility has been highlighted for Gardasil™ (Merck & Co. Inc., #9682307) in which a change of 2nd dose from month 2 to 1 can be tolerated. To facilitate a comparison of immune responses elicited to both vaccines the schedules have been synchronized, where each individual will be vaccinated at months 0, 1 & 6.

**Activity**	**Month**												
Vaccination (Cervarix™ or Gardasil™)	0	1	2	3	4	5	6	7	8	9	10	11	12
Blood taken by Vaccine Research Nurse	0	1	2	3	4	5	6	7	8	9	10	11	12
Voluntary self-taken lower vaginal sponge	0	1	2	3	4	5	6	7	8	9	10	11	12

	**Timepoint (months)**					
**GROUP**	**0**	**1 (-2 days / +2 weeks)**	**2 (+/- 1 week)**	**6 (+/- 1 week)**	**7 (+/- 1 week)**	**12 (+/- 1 week)**
**1** (n=200)	Cervarix™ **Blood sample**	Cervarix™	**Blood sample**	Cervarix™	**Samples (incl. extra blood and optional sponge)**	**Blood sample**
**2** (n=200)	Gardasil™ **Blood sample**	Gardasil™	**Blood sample**	Gardasil™	**Samples (incl. extra blood and optional sponge)**	**Blood sample**

"Samples" denotes the collection of:
- 5mL blood (clotted) at Month 0, 2, 7 and 12
- 20-30mL blood (heparinised) at Month 7 may be taken
- (Optional) self-collected lower vaginal sponge on Month 7

The above person is enrolled in this study and the 1st dose of vaccine given on ____/____/____ .

If you would like further information on this study, please contact the study organisers listed below:

Prof Elizabeth Miller Tel: 020 8327 7434
Head, Immunisation Division
CfI, Health Protection Agency

Dr Chee Yung Tel: 020 8327 7603
Consultant for Clinical Trials
CfI, Health Protection Agency

Appendix 2:

Participating Organisations and Study Personnel

Investigators

Prof Elizabeth Miller
Head – Immunisation Department
CfI, Health Protection Agency
61 Colindale Ave
London NW9 5EQ

Telephone: 0208 327 7430
Fax: 0208 200 7868

Co-ordinating Centre

Dr Simon Beddows
Scientific Advisor

Mr Nick Andrews
Statistical Advisor

Mr Tom Nichols
Trial statistician

Pauline Kaye
Data Manager

Dr Chee Yung
Consultant for Clinical Trials

Joan Vurdien
Finance Officer

Immunisation Division
Communicable Disease Surveillance Centre
Health Protection Agency
61 Colindale Avenue
London
NW9 5EQ

Tel: 0208 200 6868
Fax: 0208 200 7868

Appendix 3:

Adverse Events

Severe adverse events in post vaccination period
Any of the following local or systemic symptoms will be defined as a severe adverse event and will be reported within 24 hours by the Vaccine Research Nurse to the co-ordinating centre, at CfI or Gloucester:

LOCAL: An extensive area of redness and swelling at the site of injection which becomes indurated and involves most of the circumference of the arm.

SYSTEMIC: Fever > 39.5^{o}C within 48 hours of vaccination; anaphylaxis; bronchospasm; laryngeal oedema; generalised collapse; prolonged unresponsiveness.

Serious adverse events (SAEs)
Reporting of SAEs will be conducted in line with the relevant HPA Immunisation Department SOP.

Any of the following conditions occurring during the study period will be considered a serious event. Subjects will be asked to contact the Vaccine Research Nurse if these occur and this will then be reported within 24 hours by the Vaccine Research Nurse to the co-ordinating centre, at CfI:

- **Death**
- **Non-elective hospitalisation**
- **Any event leading to sequelae**
- **Life threatening event**

ICH GCP guidelines for reporting SAEs will be followed:

Subjects will be asked to contact their Vaccine Research Nurse if they have a severe or serious adverse event during the study period. Vaccine Research Nurses will inform the co-ordinating centre, CfI, of such events within 24 hours. The VEC SAE form will be reviewed by the medic at CfI. Action will then be taken depending on whether the SAE is deemed to be related to the vaccine or if it is considered a SUSAR
The causal relationship between severe/serious events and vaccination will be categorised by the investigators, after appropriate specialist consultation where necessary, as one of the following: related; probable; possible; remote or unrelated. Events will be graded by the clinical investigator as mild, moderate or severe.

Suspected Serious Adverse Reactions will (SSARs) be reported through the annual safety report.

All Suspected Unexpected Serious Adverse Reactions (SUSARs) will be reported by the HPA to the MHRA and the ethics committee within seven days for life threatening/fatal reactions, and within 15 days for all other severe unexpected reactions. SUSARs will be reported to the MHRA using the CIOMS (Council for International Organisations of Medical Sciences) form. The form will include the EudraCT number, CTA number, protocol number and study name.

Manufacturers will be informed of a severe or serious event using a standard adverse event report form where relation to vaccination is suspected by study medics.

Appendix 4:

Site file index

SITE FILE INDEX

A Phase IV, randomised study to evaluate the immune responses of UK adolescent girls receiving Cervarix™ or Gardasil™ Human Papillomavirus vaccines (study code **HPV CSP01**)

1. Protocol

An overview of the objectives and design of the trial, and the selection and recruitment of subjects. Details of the organisations involved and the procedures used in the study. Amendments made during the trial will be forwarded to the site for inclusion in this file.

This section will also include: **Investigator agreement**
Documents the agreement of personnel involved in the trial to conduct the trial according to the protocol – signed protocol approval forms.

2. Patient information

A copy of the documentation given to patients prior to and during the study giving information on the conduct of the trial and the safety precautions. This includes consent forms and advertisements for recruitment of subjects.

3. Product information

As licensed vaccines will be used in this study, copies of the product inserts for each vaccine to be administered will made available to the VRNs for reference

4. Trial approvals

Details of the approval of the trial by appropriate ethics and committees and coverage by indemnity agreements in the event of any trial-related incidents. This shows the trial is in compliance with the applicable regulatory requirements.

5. Drug accountability

Documents instructions needed to ensure proper storage, packaging, dispensing and disposition of trial related materials. Nobody in the practice should need to become involved in this aspect of the trial - this section is for information only.

6. Monitoring visit log

Documentation of site visits by, and findings of, the trial monitor.

7. Signature sheet

A record of all persons involved in conducting the trial who may have signed documents in the course of their involvement at this site as well as centrally to the HPA.

8. Communication for specific sites

Documentation of communication between the HPA and this site.

9. Investigator delegation list

Contact details for various possible scenarios.

This section will also include: **CVs**
Detailing the qualifications and experience of all clinical personnel involved in the trial relevant to this site.

10. Subject log

A confidential register of all the subjects in the trial at this site and their dates of vaccination. (Logged chronologically by subject number).

This section will also include: **Consent forms**
The original consent forms signed by the parent, subject (where appropriate) and Vaccine Research Nurse prior to the enrolment of the subject into the study.

Additional information
The following documents are held centrally at CfI and copies are available to personnel involved in the study on request:

- Membership lists of ethics committees
- Clinical research agreements
- Full indemnity documentation
- Vaccine tracking forms
- Study reports for ethics committees, HPA and Department of Health
- Any papers published as a result of the study

If you would like copies of any of these documents please contact Clinical Trials Admin Team, at the CfI on 020 8327 7471.

Appendix 5:

Declaration of Helsinki

WORLD MEDICAL ASSOCIATION
DECLARATION OF HELSINKI

Ethical Principles for Medical Research Involving Human Subjects
Adopted by the 18th WMA General Assembly, Helsinki, Finland, June 1964, and amended by the:
29th WMA General Assembly, Tokyo, Japan, October 1975
35th WMA General Assembly, Venice, Italy, October 1983
41st WMA General Assembly, Hong Kong, September 1989
48th WMA General Assembly, Somerset West, Republic of South Africa, October 1996
52nd WMA General Assembly, Edinburgh, Scotland, October 2000
53rd WMA General Assembly, Washington 2002 (Note of Clarification on paragraph 29 added)
55th WMA General Assembly, Tokyo 2004 (Note of Clarification on Paragraph 30 added)
59th WMA General Assembly, Seoul, October 2008

A. INTRODUCTION

1. The World Medical Association (WMA) has developed the Declaration of Helsinki as a statement of ethical principles for medical research involving human subjects**,** including research on identifiable human material and data. The Declaration is intended to be read as a whole and each of its constituent paragraphs should not be applied without consideration of all other relevant paragraphs.

2. Although the Declaration is addressed primarily to physicians, the WMA encourages other participants in medical research involving human subjects to adopt these principles.

3. It is the duty of the physician to promote and safeguard the health of patients, including those who are involved in medical research. The physician's knowledge and conscience are dedicated to the fulfilment of this duty.

4. The Declaration of Geneva of the WMA binds the physician with the words, “The health of my patient will be my first consideration,” and the International Code of Medical Ethics declares that, “A physician shall act in the patient's best interest when providing medical care.”

5. Medical progress is based on research that ultimately must include studies involving human subjects. Populations that are underrepresented in medical research should be provided appropriate access to participation in research.

6. In medical research involving human subjects, the well-being of the individual research subject must take precedence over all other interests.

7. The primary purpose of medical research involving human subjects is to understand the causes, development and effects of diseases and improve preventive, diagnostic and therapeutic interventions (methods, procedures and treatments). Even the best current interventions must be evaluated continually through research for their safety, effectiveness, efficiency, accessibility and quality.

8. In medical practice and in medical research, most interventions involve risks and burdens.

9. Medical research is subject to ethical standards that promote respect for all human subjects and protect their health and rights. Some research populations are particularly vulnerable and need special protection. These include those who cannot give or refuse consent for themselves and those who may be vulnerable to coercion or undue influence.

10. Physicians should consider the ethical, legal and regulatory norms and standards for research involving human subjects in their own countries as well as applicable international norms and standards. No national or international ethical, legal or regulatory requirement should reduce or eliminate any of the protections for research subjects set forth in this Declaration.

B. PRINCIPLES FOR ALL MEDICAL RESEARCH

11. It is the duty of physicians who participate in medical research to protect the life, health, dignity, integrity, right to self-determination, privacy, and confidentiality of personal information of research subjects.

12. Medical research involving human subjects must conform to generally accepted scientific principles, be based on a thorough knowledge of the scientific literature, other relevant sources of information, and adequate laboratory and, as appropriate, animal experimentation. The welfare of animals used for research must be respected.

13. Appropriate caution must be exercised in the conduct of medical research that may harm the environment.

14. The design and performance of each research study involving human subjects must be clearly described in a research protocol. The protocol should contain a statement of the ethical considerations involved and should indicate how the principles in this Declaration have been addressed. The protocol should include information regarding funding, sponsors, institutional affiliations, other potential conflicts of interest, incentives for subjects and provisions for treating and/or compensating subjects who are harmed as a consequence of participation in the research study. The protocol should describe arrangements for post-study access by study subjects to interventions identified as beneficial in the study or access to other appropriate care or benefits.

15. The research protocol must be submitted for consideration, comment, guidance and approval to a research ethics committee before the study begins. This committee must be independent of the researcher, the sponsor and any other undue influence. It must take into consideration the laws and regulations of the country or countries in which the research is to be performed as well as applicable international norms and standards but these must not be allowed to reduce or eliminate any of the protections for research subjects set forth in this Declaration. The committee must have the right to monitor ongoing studies. The researcher must provide monitoring information to the committee, especially information about any serious adverse events. No change to the protocol may be made without consideration and approval by the committee.

16. Medical research involving human subjects must be conducted only by individuals with the appropriate scientific training and qualifications. Research on patients or healthy volunteers requires the supervision of a competent and appropriately qualified physician or other health care professional. The responsibility for the protection of research subjects must always rest with the physician or other health care professional and never the research subjects, even though they have given consent.

17. Medical research involving a disadvantaged or vulnerable population or community is only justified if the research is responsive to the health needs and priorities of this population or community and if there is a reasonable likelihood that this population or community stands to benefit from the results of the research.

18. Every medical research study involving human subjects must be preceded by careful assessment of predictable risks and burdens to the individuals and communities involved in the research in comparison with foreseeable benefits to them and to other individuals or communities affected by the condition under investigation.

19. Every clinical trial must be registered in a publicly accessible database before recruitment of the first subject.

20. Physicians may not participate in a research study involving human subjects unless they are confident that the risks involved have been adequately assessed and can be satisfactorily managed. Physicians must immediately stop a study when the risks are found to outweigh the potential benefits or when there is conclusive proof of positive and beneficial results.

21. Medical research involving human subjects may only be conducted if the importance of the objective outweighs the inherent risks and burdens to the research subjects.

22. Participation by competent individuals as subjects in medical research must be voluntary. Although it may be appropriate to consult family members or community leaders, no competent individual may be enrolled in a research study unless he or she freely agrees.

23. Every precaution must be taken to protect the privacy of research subjects and the confidentiality of their personal information and to minimize the impact of the study on their physical, mental and social integrity.

24. In medical research involving competent human subjects, each potential subject must be adequately informed of the aims, methods, sources of funding, any possible conflicts of interest, institutional affiliations of the researcher, the anticipated benefits and potential risks of the study and the discomfort it may entail,

and any other relevant aspects of the study. The potential subject must be informed of the right to refuse to participate in the study or to withdraw consent to participate at any time without reprisal. Special attention should be given to the specific information needs of individual potential subjects as well as to the methods used to deliver the information. After ensuring that the potential subject has understood the information, the physician or another appropriately qualified individual must then seek the potential subject's freely-given informed consent, preferably in writing. If the consent cannot be expressed in writing, the non-written consent must be formally documented and witnessed.

25. For medical research using identifiable human material or data, physicians must normally seek consent for the collection, analysis, storage and/or reuse. There may be situations where consent would be impossible or impractical to obtain for such research or would pose a threat to the validity of the research. In such situations the research may be done only after consideration and approval of a research ethics committee.

26. When seeking informed consent for participation in a research study the physician should be particularly cautious if the potential subject is in a dependent relationship with the physician or may consent under duress. In such situations the informed consent should be sought by an appropriately qualified individual who is completely independent of this relationship.

27. For a potential research subject who is incompetent, the physician must seek informed consent from the legally authorized representative. These individuals must not be included in a research study that has no likelihood of benefit for them unless it is intended to promote the health of the population represented by the potential subject, the research cannot instead be performed with competent persons, and the research entails only minimal risk and minimal burden.

28. When a potential research subject who is deemed incompetent is able to give assent to decisions about participation in research, the physician must seek that assent in addition to the consent of the legally authorized representative. The potential subject's dissent should be respected.

29. Research involving subjects who are physically or mentally incapable of giving consent, for example, unconscious patients, may be done only if the physical or mental condition that prevents giving informed consent is a necessary characteristic of the research population. In such circumstances the physician should seek informed consent from the legally authorized representative. If no such representative is available and if the research cannot be delayed, the study may proceed without informed consent provided that the specific reasons for involving subjects with a condition that renders them unable to give informed consent have been stated in the research protocol and the study has been approved by a research ethics committee. Consent to remain in the research should be obtained as soon as possible from the subject or a legally authorized representative.

30. Authors, editors and publishers all have ethical obligations with regard to the publication of the results of research. Authors have a duty to make publicly available the results of their research on human subjects and are accountable for the completeness and accuracy of their reports. They should adhere to accepted guidelines for ethical reporting. Negative and inconclusive as well as positive results should be published or otherwise made publicly available. Sources of funding, institutional affiliations and conflicts of interest should be declared in the publication. Reports of research not in accordance with the principles of this Declaration should not be accepted for publication.

C. ADDITIONAL PRINCIPLES FOR MEDICAL RESEARCH COMBINED WITH MEDICAL CARE

31. The physician may combine medical research with medical care only to the extent that the research is justified by its potential preventive, diagnostic or therapeutic value and if the physician has good reason to believe that participation in the research study will not adversely affect the health of the patients who serve as research subjects.

32. The benefits, risks, burdens and effectiveness of a new intervention must be tested against those of the best current proven intervention, except in the following circumstances:

- The use of placebo, or no treatment, is acceptable in studies where no current proven intervention exists; or
- Where for compelling and scientifically sound methodological reasons the use of placebo is necessary to determine the efficacy or safety of an intervention and the patients who receive placebo or no treatment will not be subject to any risk of serious or irreversible harm. Extreme care must be taken to avoid abuse of this option.

33. At the conclusion of the study, patients entered into the study are entitled to be informed about the outcome of the study and to share any benefits that result from it, for example, access to interventions identified as beneficial in the study or to other appropriate care or benefits.

34. The physician must fully inform the patient which aspects of the care are related to the research. The refusal of a patient to participate in a study or the patient's decision to withdraw from the study must never interfere with the patient-physician relationship
.

35. In the treatment of a patient, where proven interventions do not exist or have been ineffective, the physician, after seeking expert advice, with informed consent from the patient or a legally authorized representative, may use an unproven intervention if in the physician's judgement it offers hope of saving life, re-establishing health or alleviating suffering. Where possible, this intervention should be made the object of research, designed to evaluate its safety and efficacy. In all cases, new information should be recorded and, where appropriate, made publicly available.

Appendix 6:

Protocol Approval Forms

INVESTIGATOR PROTOCOL APPROVAL FORM

Protocol title: A Phase IV, randomised study to evaluate the immune responses of UK adolescent girls receiving Cervarix^{TM} or Gardasil^{TM} Human Papillomavirus vaccines (study code **HPV CSP01**)

I have read and approved the attached protocol. I shall comply with its contents and I accept the investigator responsibilities as defined in the 1996 ICH GCP guidelines.

____________________________ ____/____/____

Investigator Signature Date

____________________________ ____________________________

Investigator Name Title

Address

____

____

____

GENERAL PRACTITIONER PROTOCOL APPROVAL FORM

Protocol title: A Phase IV, randomised study to evaluate the immune responses of UK adolescent girls receiving Cervarix^{TM} or Gardasil^{TM} Human Papillomavirus vaccines (study code **HPV CSP01**)

The attached protocol has been read and approved according to our practice procedure. I confirm, on behalf of the GPs at the Surgery named below, that this study has been approved to take place in the Surgery such that patients may be approached to participate by Health Protection Agency Vaccine Research Nurse.

The Surgery will be provided with a site file (see index pages in Appendix 4). Included there is a list of responsibilities. The GPs at the Surgery are willing to comply with these, such that we:

- Ensure appropriate management of patients involved in the study according to practice protocol for vaccination
- Undertake to report, where possible, all serious / severe adverse events to the investigators or Vaccine Research Nurses (VRNs) as quickly as possible so that appropriate action can be taken
- Allow VRNs to vaccinate at surgery and have access to patient records

_____________________________   ______/_____/____

Signature of Surgery representative   Date

_____________________________   ___________________________

Name   Title

Surgery address/ stamp

____

____
